# Consistent inverse correlation between DNA methylation of the first intron and gene expression across tissues and species

**DOI:** 10.1186/s13072-018-0205-1

**Published:** 2018-06-29

**Authors:** Dafni Anastasiadi, Anna Esteve-Codina, Francesc Piferrer

**Affiliations:** 1 Institute of Marine Sciences (ICM-CSIC), Passeig Marítim de la Barceloneta, 37-49, 08003 Barcelona, Spain; 2 CNAG-CRG, Center for Genomic Regulation (CRG), Barcelona Institute of Science and Technology (BIST), Baldiri i Reixac 4, 08028 Barcelona, Spain; 3 Universitat Pompeu Fabra (UPF), Barcelona, Spain

**Keywords:** DNA methylation, Gene expression, First intron, Regulation, Gene features

## Abstract

**Background:**

DNA methylation is one of the main epigenetic mechanisms for the regulation of gene expression in eukaryotes. In the standard model, methylation in gene promoters has received the most attention since it is generally associated with transcriptional silencing. Nevertheless, recent studies in human tissues reveal that methylation of the region downstream of the transcription start site is highly informative of gene expression. Also, in some cell types and specific genes it has been found that methylation of the first intron, a gene feature typically rich in enhancers, is linked with gene expression. However, a genome-wide, tissue-independent, systematic comparative analysis of the relationship between DNA methylation in the first intron and gene expression across vertebrates has not been explored yet.

**Results:**

The most important findings of this study are: (1) using different tissues from a modern fish, we show a clear genome-wide, tissue-independent quasi-linear inverse relationship between DNA methylation of the first intron and gene expression. (2) This relationship is conserved across vertebrates, since it is also present in the genomes of a model pufferfish, a model frog and different human tissues. Among the gene features, tissues and species interrogated, the first intron’s negative correlation with the gene expression was most consistent. (3) We identified more tissue-specific differentially methylated regions (tDMRs) in the first intron than in any other gene feature. These tDMRs have positive or negative correlation with gene expression, indicative of distinct mechanisms of tissue-specific regulation. (4) Lastly, we identified CpGs in transcription factor binding motifs, enriched in the first intron, the methylation of which tended to increase with the distance from the first exon–first intron boundary, with a concomitant decrease in gene expression.

**Conclusions:**

Our integrative analysis clearly reveals the important and conserved role of the methylation level of the first intron and its inverse association with gene expression regardless of tissue and species. These findings not only contribute to our basic understanding of the epigenetic regulation of gene expression but also identify the first intron as an informative gene feature regarding the relationship between DNA methylation and gene expression where future studies should be focused.

**Electronic supplementary material:**

The online version of this article (10.1186/s13072-018-0205-1) contains supplementary material, which is available to authorized users.

## Background

DNA methylation is one of the main epigenetic mechanisms for the regulation of gene expression [1]. Under the so-called standard model of gene expression regulation, methylation of cytosine–guanine dinucleotides (CpGs) in the promoter regions of genes has received the most attention since it is generally associated with repression of transcription, either directly, by blocking the access of transcription factors (TFs), or indirectly, by recruiting other repressive proteins with methyl-binding domains [2, 3]. Regions rich in CpGs that typically span 200–1000 bp are called CpG islands (CGI), usually remain unmethylated, overlap with gene promoters and are associated with gene transcription regulation [4, 5].

Nevertheless, recent studies in human tissues reveal that methylation of the region downstream of the transcription start site (TSS) is highly informative of gene expression.

Thus, in addition to promoters, enhancers also bind TFs, interact with the promoter, and exhibit widespread hypo-methylation during development [6] and dynamic changes during oncologic transformation [7, 8]. Also, studies using mammalian cells have shown differences in methylation levels between the first exon and the rest of exons and, further, gene expression levels are better inversely correlated with the methylation of the first exon than with that of the promoter [9]. Furthermore, between gene body methylation and gene expression, a positive correlation has been demonstrated [10]. These studies suggest that DNA methylation of distal or intragenic regulatory elements with different degrees of CpG density are involved in the regulation of gene expression and that DNA methylation has dual roles, both inhibitory and permissive, depending on the genomic region.

Differences in the contribution of DNA methylation to gene expression regulation among distinct genomic features are also evident in the so-called tissue-specific differentially methylated regions (tDMRs), which are located both upstream and downstream of the transcription start site [11]. These tDMRs contain binding sites for different TFs and overlap with regions of variable CpG density, and although their hypo-methylation is thought to be related to tissue-specific functions, they can also exhibit positive or negative correlation with gene expression levels [11, 12].

Recent comparative epigenomic studies using non-model organisms have shown that epigenetic divergence follows the genetic phylogenetic patterns across species [13, 14]. Thus, across vertebrates there are global differences in the methylation content of warm-blooded versus cold-blooded species [15]. Research in epigenetics of non-model vertebrates including fish [16–22], birds [23, 24] and mammals [13, 25–30] is generally undertaken with the main objective to correlate DNA methylation patterns with a specific phenotypic trait. However, a genome-wide, tissue-independent, systematic comparative analysis of the relationship between DNA methylation in defined and distinct genomic features and gene expression across vertebrates has not been explored yet.

To address these questions, here we used the European sea bass (*Dicentrarchus labrax*), a modern teleost and one of the fish species with more genomic resources available [31, 32]. To account for the possible influence of cellular diversity when compared to cell lines, we selected the muscle, where myocytes clearly dominate, hence a tissue of very low cellular diversity, and the adult testis, where different types of somatic and germ cells coexist, thus a tissue of high cellular diversity. We constructed reduced representation bisulfite sequencing (RRBS) libraries to measure the genome-wide DNA methylation and RNA-seq libraries to measure gene expression levels. We determined the relationship between DNA methylation and transcriptomic profiles in different genomic features including not only promoters but also introns and exons. We found that a clear inverse correlation between DNA methylation and gene expression is present in the first intron. Results were contrasted not only with results obtained in mammals but also with those obtained in other vertebrates, including the model fish *Tetraodon nigroviridis* (pufferfish) and model frog *Xenopus tropicalis*. Then, we investigated the functional properties of this relationship and we identified CpGs in TF-binding motifs enriched in the first intron, which were close to the beginning of first intron and were indicators of gene expression. Lastly, we detected tDMRs between the two tissues of different transcriptomic complexity which correlated with gene expression.

## Results

### The relation of DNA methylation and gene expression depends on the gene feature

**Fig. 1:**
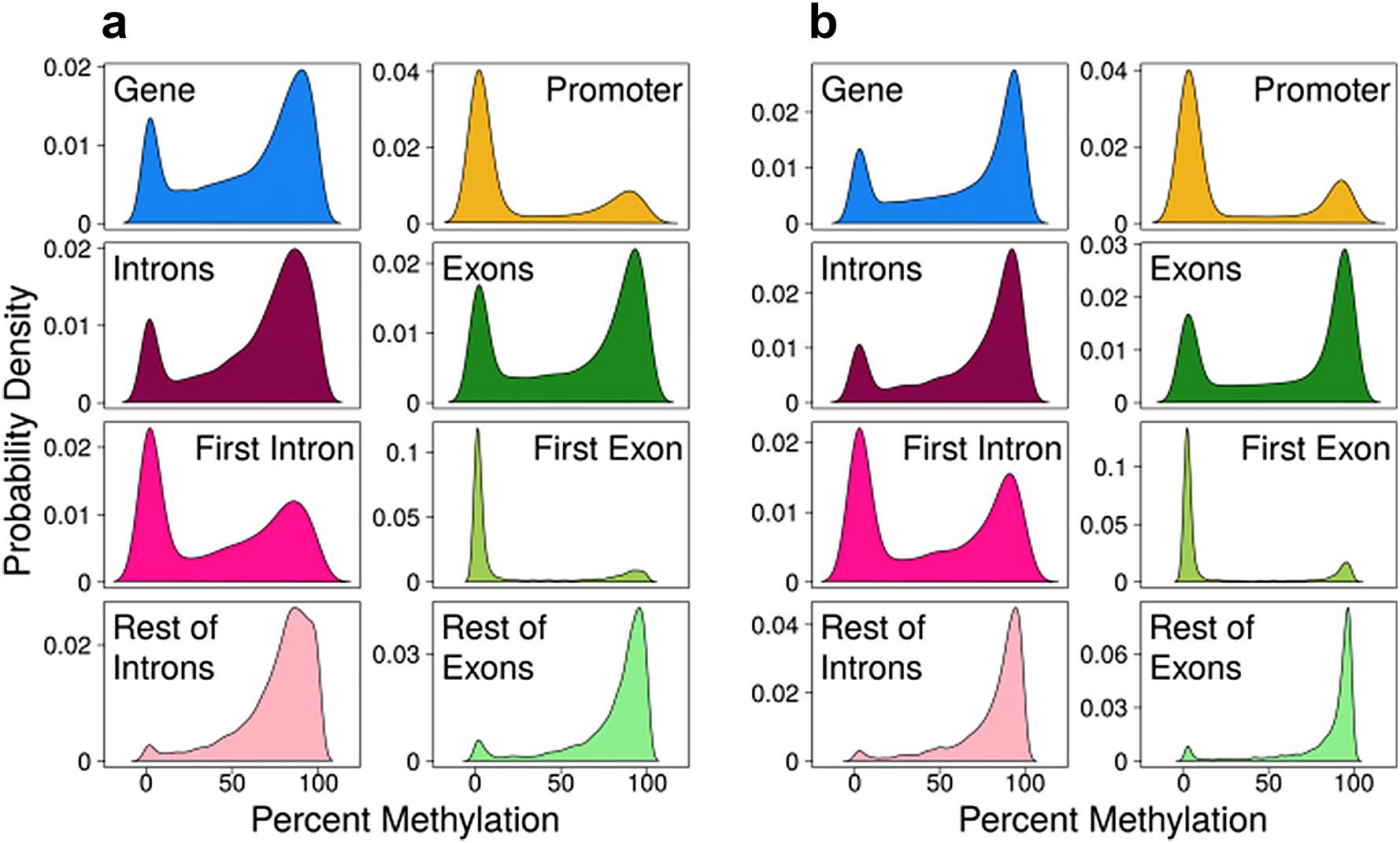
DNA methylation per gene in gene features in muscle (**a**) and in testis (**b**). Kernel density plots for DNA methylation in genes (*n* = 15,456), promoters (− 1000 bp from the transcription start site; *n* = 5034), all introns (*n* = 9184) and all exons (*n* = 12,317). Separation of exons in first exon (*n* = 5790) and rest of exons (*n* = 8798) and of introns in first intron (*n* = 4387) and rest of introns (*n* = 5646)

In whole genes, DNA methylation patterns followed a bimodal distribution, with high (> 80%) or low (~ 10%) levels of DNA methylation in the majority of CpG sites in both muscle (Fig. 1a) and testis (Fig. 1b). Separating the whole gene in specific gene features exposed distinct patterns. A similar bimodal DNA methylation pattern was observed in introns and exons. However, in promoters, most of CpG sites were unmethylated. In addition, by partitioning data from exons into first exon and the rest of exons, a contrasting pattern was revealed. The majority of unmethylated cytosines were restricted to the first exon and methylated cytosines almost exclusively localized in the rest of exons, while the peak of unmethylated cytosines was sharper in the first exon than in the promoter. Likewise, partitioning the introns showed a majority of highly methylated cytosines in all except the first intron. In the first intron, the distribution was still bimodal but skewed toward the unmethylated sites and smoother than in the first exon and the promoter (Fig. 1a, b). The distribution of DNA methylation in specific gene features was similar in liver and spleen, for which one RRBS library per tissue was also constructed (Additional file 1: Fig. S1). The RRBS libraries for liver and spleen were constructed for a preliminary study and were not further analyzed since no biological replicates were sequenced. Thus, regardless of tissue and cellular diversity, the majority of CpG sites were unmethylated in the promoter and first exon and, to a lesser degree, also in the first intron.

**Fig. 2:**
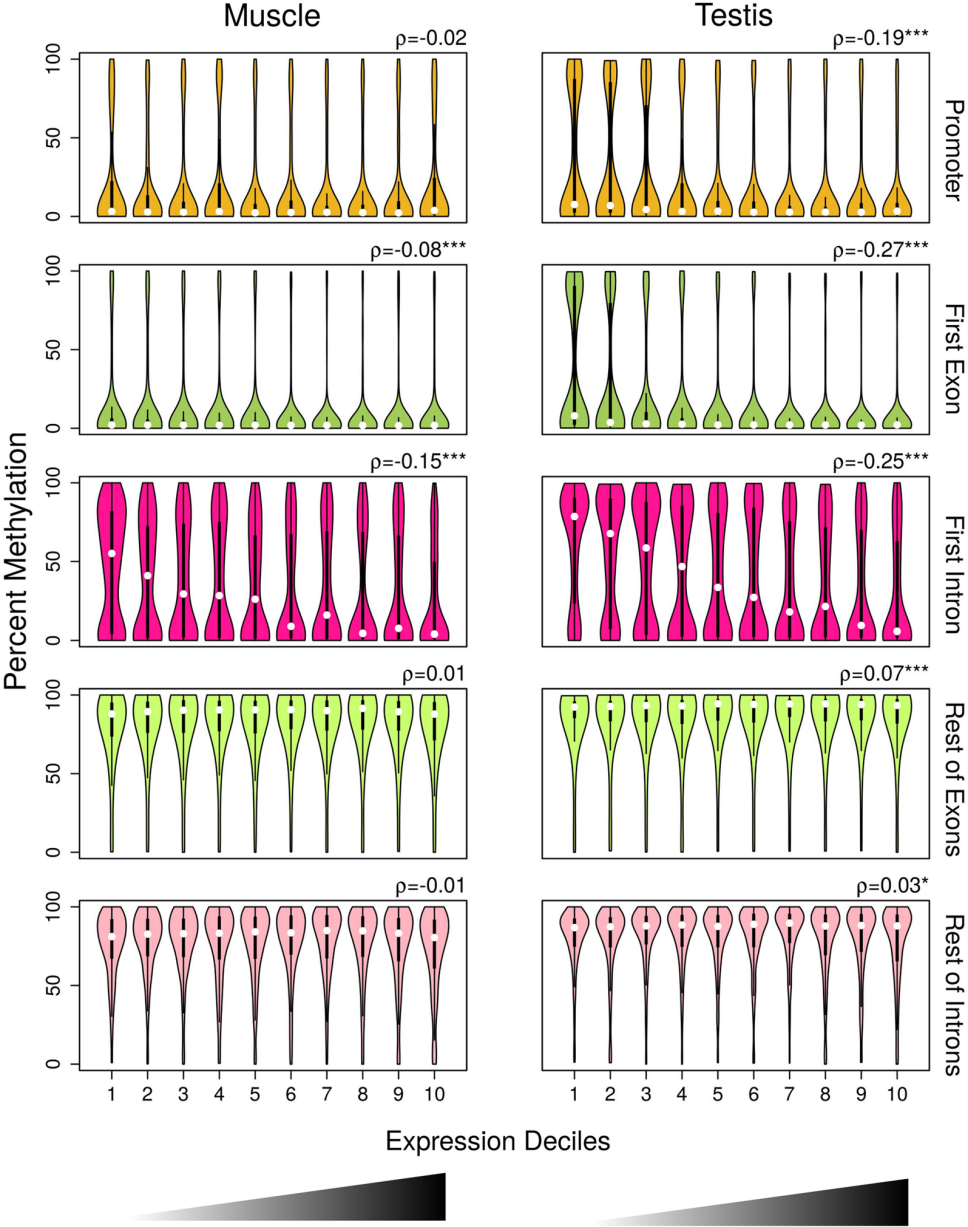
DNA methylation in gene features by expression deciles in muscle and in testis. Violin plots of DNA methylation in promoter (muscle, *n* = 2745; testis, *n* = 3345), first exon (muscle, *n* = 3537; testis, *n* = 4064), first intron (muscle, *n* = 2801; testis, *n* = 3122), rest of exons (muscle, *n* = 5523; testis, *n* = 6398) and rest of introns (muscle, *n* = 4043; testis, *n* = 4897) divided into deciles based on increasing ranking of gene expression measured as log_2_-transformed count per million (cpm) values. Box plots with rotated kernel density plots at both sides indicate the interquartile range, and white central dots the median of the distribution. Correlations between DNA methylation and gene expression were measured using Spearman’s rank correlation coefficient (*ρ*), and the significance levels are reported as follows: *p < 0.05; ***p < 0.001

In order to relate the gene expression levels with the DNA methylation levels of specific gene features, we divided the gene expression levels in deciles based on the increasing distribution of log_2_-transformed copy million number (cpm) values. In muscle, median DNA methylation levels were low regardless of gene expression in promoter and first exon (Fig. 2). In the first exon, there was also a weak but significant negative correlation of DNA methylation with gene expression (Spearman’s rank correlation coefficient [*ρ*] = − 0.08, *p* value < 0.001). By contrast, in the first intron DNA methylation levels decreased with increasing expression levels (Fig. 2) and there was the strongest among gene features negative correlation of DNA methylation with gene expression (*ρ* = − 0.15, *p* < 0.001). In the rest of exons and introns, DNA methylation levels were high independently of gene expression and there were no significant correlations of DNA methylation with gene expression.

In testis, in the promoter and first exon, median DNA methylation levels were also low in all expression deciles (Fig. 2). However, in the genes belonging to the first and second expression deciles, there was significantly more variance in the DNA methylation levels in comparison with the muscle (ANOVA on residuals followed by Tukey’s HSD; *p* adjusted < 0.001) and also compared to the rest of expression deciles (*p* < 0.001). In the testis, significant negative correlations of DNA methylation with gene expression were evident in the promoter (*ρ* = − 0.19; *p* < 0.001), first exon (*ρ* = − 0.27; *p* < 0.001) and first intron (*ρ* = − 0.25; *p* < 0.001). The negative correlation in the first intron was stronger than the one in the promoter, similarly to what was observed in muscle, but slightly weaker than in the first exon, in contrast to what was observed in muscle. In the rest of exons and in the rest of introns, median DNA methylation levels were high regardless of gene expression levels and the correlations were weakly positive, although significant (*p* < 0.05). Thus, gene expression was clearly inversely correlated with DNA methylation levels across the two tissues only in the first intron.

This inverse relationship became more evident when we only considered genes at the extremes of the expression range in both tissues. For example, genes with low expression (members of the first and second expression deciles) or with high expression (members of the ninth and tenth expression deciles) exhibited similar and clearer patterns of DNA methylation (Additional file 1: Fig. S2).

### The inverse relationship of DNA methylation and gene expression is present in other vertebrate genomes

**Fig. 3:**
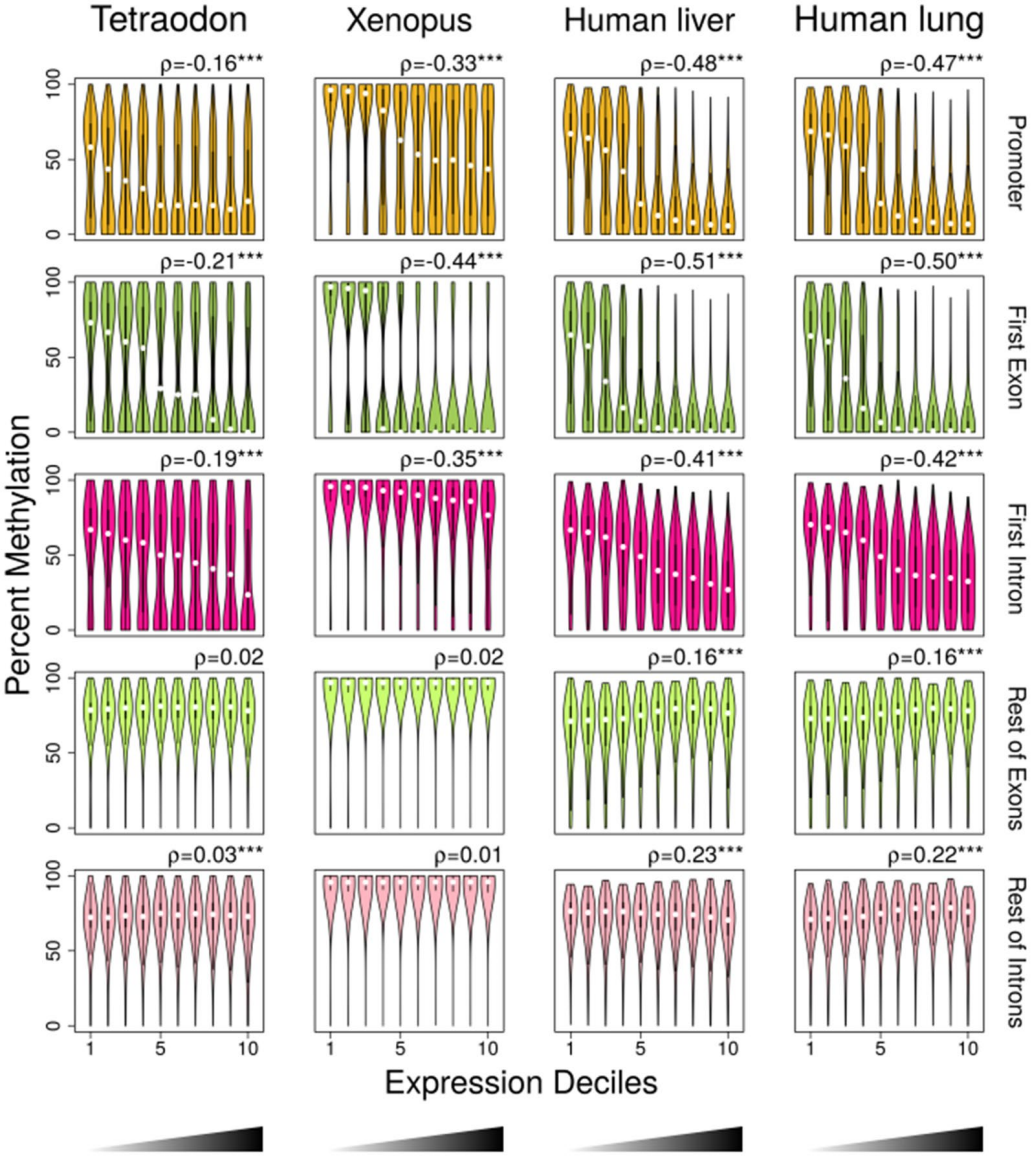
Violin plots of DNA methylation in promoter (Tetraodon, *n* = 12,896; Xenopus, *n* = 12,704; human liver, *n* = 22,680; human lung, *n* = 23,012), first exon (Tetraodon, *n* = 11,887; Xenopus, *n* = 10,361; human liver, *n* = 20,383; human lung, *n* = 20,704), first intron (Tetraodon, *n* = 11,420; Xenopus, *n* = 12,202; human liver, *n* = 20,029; human lung, *n* = 20,757), rest of exons (Tetraodon, *n* = 12,618; Xenopus, *n* = 12,662; human liver, *n* = 18,961; human lung, *n* = 19,331) and rest of introns (Tetraodon, *n* = 11,840; Xenopus, *n* = 11,905; human liver, *n* = 16,930; human lung, *n* = 17,007) divided into deciles based on increasing ranking of gene expression. Box plots with rotated kernel density plots at both sides indicate the interquartile range, and white central dots the median of the distribution. Correlations between DNA methylation and gene expression were measured using Spearman’s rank correlation coefficient (*ρ*), and the significance levels are reported as follows: ***p < 0.001

In order to investigate this inverse correlation in other vertebrate species, we, then, used whole-genome bisulfite sequencing (WGBS) and RNA-seq data from a pufferfish, whole *Tetraodon nigroviridis* [20] (NCBI’s Gene Expression Omnibus [33]; GEO with accession number GSE19824), from a frog, *Xenopus tropicalis* [34] (GEO with accession number GSE67974) and from human liver and lung [35] (GEO with accession number GSE70091). WGBS data of *Xenopus* were obtained from gastrula stage 10.5 and RNA-seq data from gastrula stage 11, therefore during development. In the promoters and first exons of these species, DNA methylation showed a decreasing relationship with gene expression in the first three to four deciles and then remained low in the deciles of higher expression (Fig. 3). The correlation of gene expression with DNA methylation was negative in all cases. In the first intron, DNA methylation was decreasing with the expression decile in all vertebrate datasets tested. In contrast, in an invertebrate species, *Ciona intestinalis*, [20] (GEO with accession number GSE19824), the correlations were always positive and significant in promoters, first exons and first introns (Additional file 1: Fig. S3).

### The DNA methylation of TF-binding motifs located at the beginning of the first intron is informative of gene expression

Next, we focused exclusively on the first intron and searched for potential enrichment of specific TF-binding motifs associated with the identified negative correlation of gene expression with DNA methylation. This analysis was performed using sequences of ± 50 bp from the CpGs with methylation values in the first introns of expressed genes, i.e., the genes of Fig. 2. This distance was chosen to encompass the maximum TF-binding motif length (31 nucleotides [36]) and an arbitrary + 20 nucleotides more. The objective was to identify TF-binding motifs that were independent of the tissue under question; therefore, after performing the enrichment compared to input shuffled sequences, we selected only the 10 TF-binding motifs that were enriched in both muscle and testis (Additional file 1: Fig. S4).

**Fig. 4:**
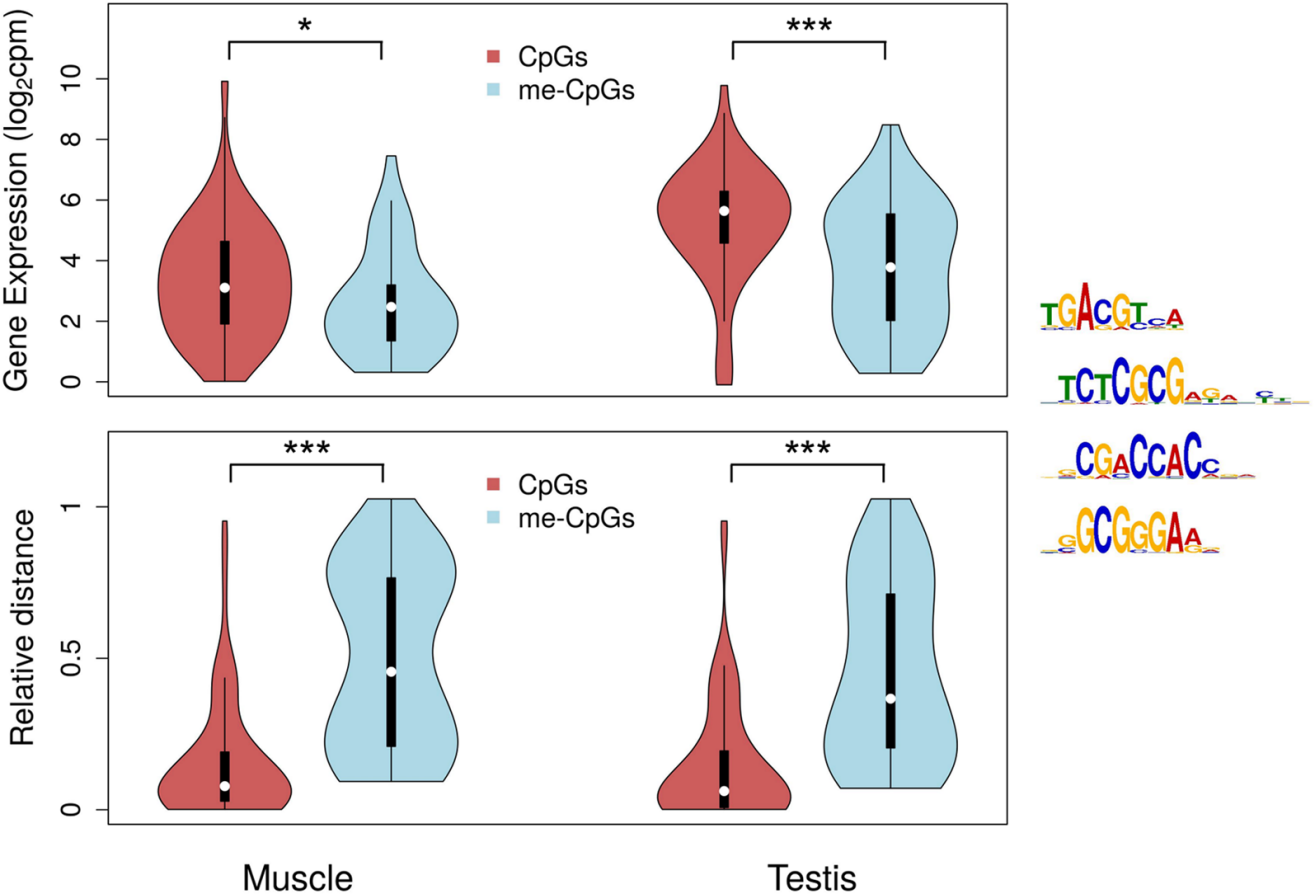
CpGs in enriched transcription factor (TF) binding sites of the first intron. CpGs were classified as unmethylated (below the first quartile of the total distribution; CpGs, dark red) or methylated (above the third quartile of the total distribution; me-CpGs, light blue) for muscle and for testis. The expression of genes measured as log_2_-transformed count per million (cpm) values is shown in the upper panel depending on the type of CpGs these genes contained in their first intron. In the lower panel, the relative distance of the CpGs and me-CpGs from the first exon-first intron boundary was calculated as distance from nucleotide 0 (bp)/width of the intron (bp). The sequences of the four enriched TF-binding motifs that contained CpGs are also shown. The Wilcoxon rank sum test with continuity correction was used to test for statistical differences of gene expression and relative distance between CpGs and me-CpGs, which are reported with the following equivalence: ***p < 0.001; *p < 0.05

The methylation status of the CpGs inside TF-binding motifs may directly affect the binding affinity. Therefore, we then focused on the 4 TFs, among the 10 enriched, in the binding motifs of which CpGs were present: CREB1, ZBTB33, ZBTB7A and E2F4. The first introns of muscle and testis were, then, screened for these 4 specific motifs, and the methylation status of the target CpGs was identified (Fig. 4). The CpGs were classified as unmethylated if their methylation was below the first quartile of the total distribution or as methylated if their methylation was above the third quartile of the total distribution for each tissue. The expression of genes with unmethylated CpGs in the target TF-binding motifs was significantly higher than the expression of genes with methylated CpGs in muscle (one-sided Wilcoxon rank sum test with continuity correction; *W* = 1432.5, *p* = 0.0171) and in testis (*W* = 1552, *p* = 0.0003). In addition, the unmethylated CpGs were located closer to the first exon–first intron boundary than the methylated CpGs in both muscle (*W* = 705, *p* = 1.222^−13^) and testis (*W* = 738, *p* = 3.887^−12^). Analysis of covariance revealed a significant effect of the interaction between relative distance of the CpG and methylation status on gene expression in both muscle (*F* = 6.264, *p* = 0.013; Table 1) and testis (*F* = 5.781, *p* = 0.018; Table 1).

**Table 1:** Effects on gene expression of the methylation status and the relative distance of the CpGs inside the four transcription factor binding motifs enriched in the first introns

Tissue	Factors	SS	*d.f.*	*F* value	*p* value
Muscle	Relative distance	5.18	1	1.872	0.173
	Methylation status	10.76	1	3.885	0.051
	Interaction of relative distance with methylation status	17.35	1	6.264	*0.013*
	Residuals	387.65	140		
Testis	Relative distance	7.31	1	1.855	0.176
	Methylation status	121.86	1	30.900	*0.000*
	Interaction of relative distance with methylation status	22.8	1	5.781	*0.018*
	Residuals	540.28	137		

The effects were tested using analysis of covariance, and the statistically significant ones are shown in italics

*d.f.* degrees of freedom, *SS* sums of squares

### The DNA methylation of the first intron associates with the upstream features and is independent of its length

**Fig. 5:**
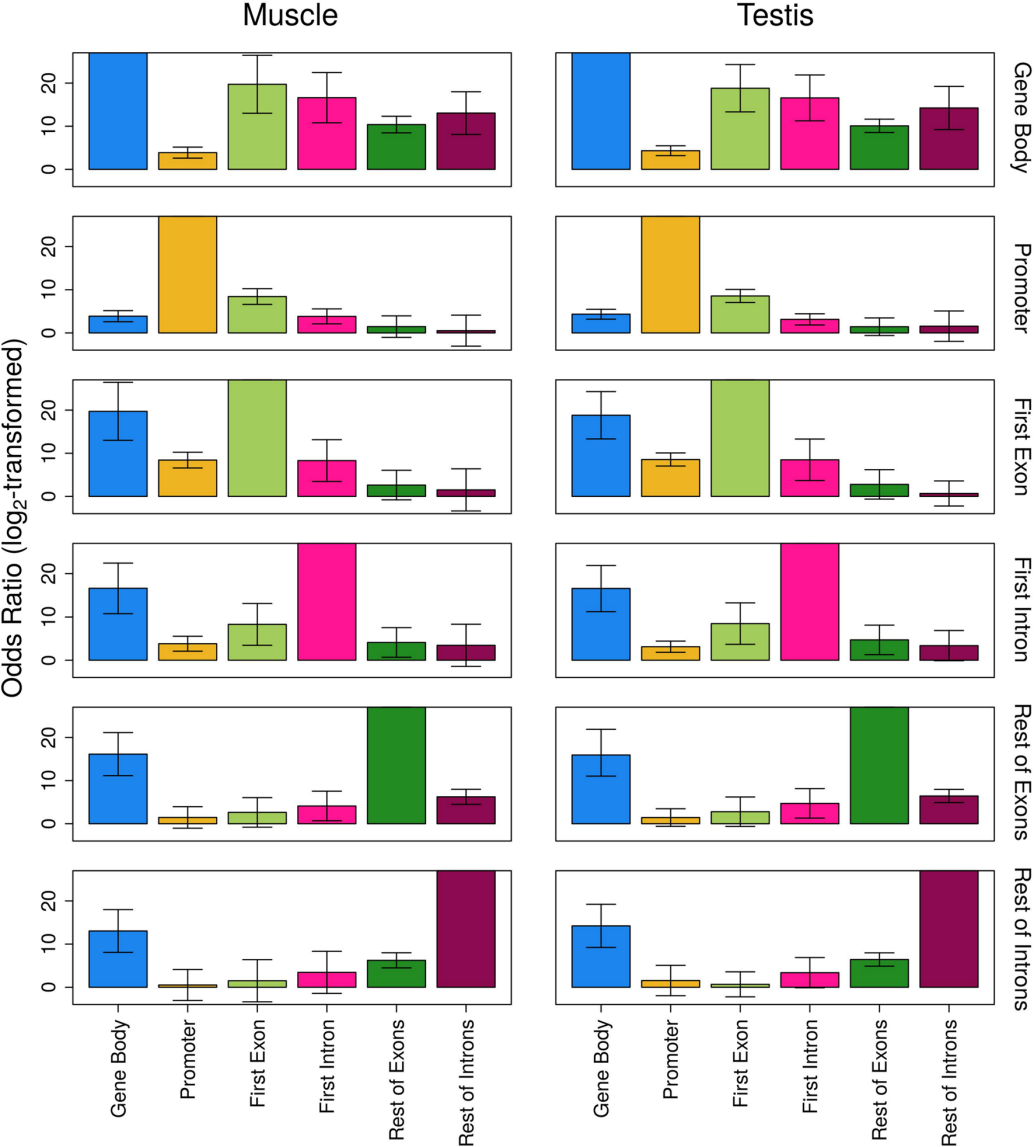
Association of DNA methylation between pairs of gene features as measured by odds ratio. A gene feature was considered methylated if DNA methylation > 90% and unmethylated if DNA methylation < 10%. The odds ratio (OR) indicates the pairwise association between the methylation states of the gene features of interest, including gene body (all exons and introns), promoter, first exon, first intron, rest of exons and rest of introns in muscle and testis. The odds ratio is represented as log_2_-transformed values, and the bars indicate the 99.9% confidence intervals based on the Wald approximation. For the associations of gene feature A versus gene feature A, values were set to maximum and confidence intervals are not applicable

In order to test whether there is association of the methylation state of two gene features, we calculated the odds ratio (OR) as representative of the odds that a gene feature A is also methylated when gene feature B is methylated. In both tissues, there was strong evidence for statistically significant association of the DNA methylation of the promoter, the first exon and the first intron (Fig. 5), since the 99.9% confidence intervals (CIs) were far from overlapping the value 1. The gene body methylation, including all exons and introns of a gene, showed strong association with the DNA methylation of all gene features tested, while the methylation of the rest of exons and the rest of introns was also associated. In testis, the methylation of the first intron was associated with the methylation of the rest of exons as well.

Since DNA methylation occurs in the CpG context, we wanted to exclude biases potentially affecting our results regarding the importance of the first intron for gene expression, mainly the CpG density in the gene features of interest and the length of the first intron. To address potential CpG density bias, we looked to the distribution of DNA methylation as a function of CpG density for the promoter, first exon and first intron, for all genes across the expression deciles. The CpG density was higher in the first exon, followed by the promoter and the first intron (pairwise Wilcoxon rank sum tests with continuity correction, *p* < 2.2^−16^ in all cases; Additional file 1: Fig. S5). The first intron showed a less dynamic range of CpG density and a more uniform distribution of the DNA methylation. To contemplate first intron length’s bias, we divided the dataset of genes in four quartiles according to the distribution of the length of their first intron. The correlation between DNA methylation and gene expression was negative independently of the length in both tissues, even in the fourth quartile of intron length that consisted of introns with median length of 18,746 bp in the muscle and 25,989 bp in the testis (Additional file 1: Table S1).

To further decipher the relationship of gene expression with DNA methylation in the first intron, we focused only on the extreme situations. Therefore, we selected the genes with the lowest (expression deciles 1 and 2) and the highest (deciles 9 and 10) expression and with mean DNA methylation below 10% or above 90% (Additional file 1: Fig. S6). The vast majority of the highest expressed genes in both tissues had DNA methylation below 10% (89.5% of genes in muscle and 84.9% in testis). However, in the lowest expressed genes, DNA methylation was more equally distributed to the two extremes, with the 75.2% of genes in the muscle and the 48.2% of genes in the testis having less than 10% methylation, and the 24.8% of genes in the muscle and the 51.8% of genes in the muscle having more than 90% methylation.

### More genes contain tDMRs in their first intron than in other gene features

**Fig. 6:**
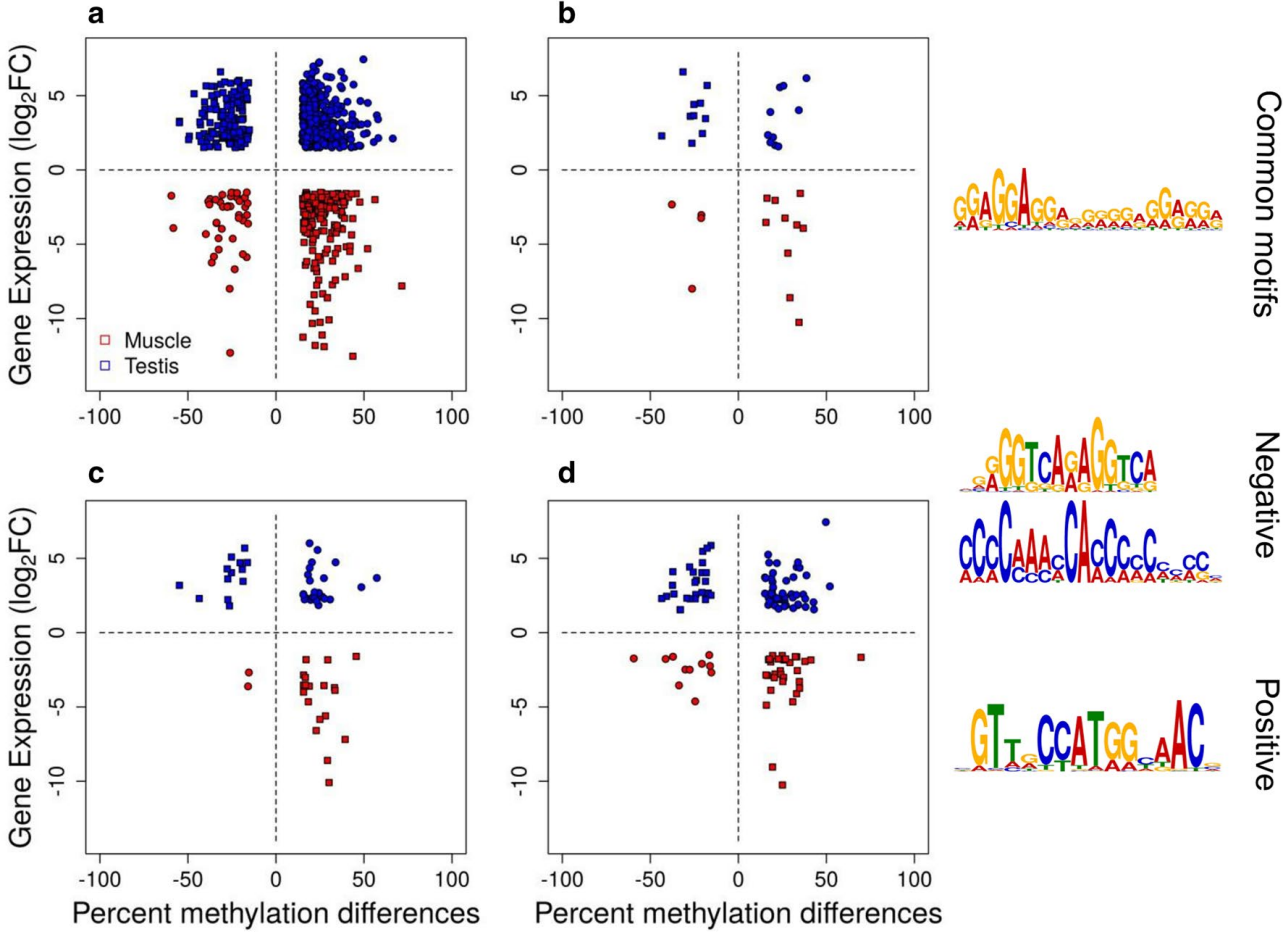
Differentially expressed genes with differentially methylated regions (tDMRs) between tissues. tDMRs overlap with the gene body and/or  4 kb upstream from the transcription start site or downstream of the 3’ UTR (**a**, *n* = 1044), the promoter (**b**, *n* = 47), the first exon (**c**, *n* = 75) or the first intron (**d**, *n* = 187). Positive (circles) and negative (boxes) correlation is shown for up-regulated genes in muscle (red) and up-regulated genes in testis (blue). Hyper-methylated tDMRs and up-regulated DEGs in testis (blue circles), hypo-methylated tDMRs and up-regulated DEGs in testis (blue squares), hypo-methylated tDMRs and up-regulated DEGs in muscle (red squares) and hyper-methylated tDMRs and up-regulated DEGs in muscle (red circles). Differentially expressed were considered the genes with log_2_ fold change > |1.5| and false discovery rate < 0.05. tDMRs were defined as regions showing more than 15% methylation difference between tissues and *q* value < 0.001, with a minimum number of 5 CpGs and 3 differentially methylated cytosines (DMCs), where a DMC showed more than 15% methylation difference between tissues. Transcription factor binding motifs present in the tDMRs of gene bodies and/or ± 4 kb that are common between positive and negative correlation of DNA methylation with gene expression or that are correlation specific (negative or positive)

In general, there were distinct patterns between the tissue-specific genes and the non-tissue-specific genes that were obvious from the gene expression data. Therefore, we focused on tDMRs between testis and muscle and explored the relationships between differential DNA methylation and differences in gene expression. Both directions of correlation were evident between DNA methylation and gene expression. There was strong negative correlation (*ρ* = − 0.73, *p* < 0.001) for up-regulated genes, in either testis or muscle that contained hypo-methylated tDMRs inside their gene body or 4 kb upstream of the TSS or downstream of the 3′ UTR (squares in Fig. 6). Nonetheless, there was also weaker positive correlation (*ρ* = 0.26, *p* < 0.001) for up-regulated genes, in either testis or muscle that contained hyper-methylated tDMRs (circles in Fig. 6). The same two categories of genes showing either negative or positive correlation between DNA methylation and gene expression were obvious in genes that contained tDMRs only in the promoter, first exon or first intron (Fig. 6). There were 187 genes that contained tDMRs in their first intron, while there were fewer genes that contained tDMRs in their promoter (47) and the first exon (75). The majority of the tDMRs in the first intron were located in proximity to the first exon-first intron boundary (Additional file 1: Fig. S7).

Next, we scanned the tDMRs present inside genes or 4 kb upstream of the TSS or downstream of the 3’ UTR for TF-binding sites in an attempt to identify features that characterize the type of correlation. The binding motif of the TF ZNF263 was common between the tDMRs associated with genes showing either positive or negative correlation. However, there were also correlation-specific motifs in each case, with the positive correlation-specific motif for binding of the regulatory factor X3 (RFX3) and two negative correlation-specific motifs for binding of the nuclear receptor subfamily 2 group C member 2 (NR2C2) and the Ras-responsive element binding protein 1 (RREB1; Fig. 6).

## Discussion

In this study, we present an inverse correlation of the DNA methylation in the first intron and gene expression, which is conserved in tissues with different levels of cellular complexity and across vertebrates. Furthermore, we detect CpGs in enriched TF-binding sites close to the first exon–first intron boundary indicative of gene expression and tDMRs the methylation of which correlates with gene expression.

Recent studies on the relationship between DNA methylation and gene expression have revealed a key role for the region from + 0.5 to + 2.5 kb downstream of the TSS in transcriptional regulation of different human tissues [37, 38]. In addition, the methylation of the first exon was shown in mammalian cell lines to be negatively correlated to gene expression, in a more pronounced way than the promoter region [9]. In parallel, the methylation of the first intron has been shown by functional studies to have both positive and negative correlation with gene expression in specific genes in cancer cell lines, fetal and adult tissues [39], CD4+ cell lines isolated from mice [40], during lineage specification in T cells [41], in multiple myeloma cell lines [42], in leukocytes of patients with schizophrenia [43] and in blood samples isolated from children [44]. Furthermore, it has been suggested that the first intron contains distinct properties from the rest of introns and that is linked to transcriptional regulation [45, 46]. These properties may be linked to the closer proximity of the first intron to the TSS, since in many species the CpG-rich regions expand from the CGI promoter to the surrounding sequences [47]. Other active chromatin marks are enriched in conserved parts of the human first intron [48]. However, to the best of our knowledge, an association at the genome-wide scale of the methylation of the first intron as an outlined gene feature has not been demonstrated to date.

In two complex tissues of a phylogenetically distant species, we present the same pattern previously observed in humans. In the European sea bass genome, the median methylation of the first intron showed, consistently in both tissues, the most clear inverse relationship with gene expression among all gene features. We further show that the inverse relationship between DNA methylation of the first intron and gene expression is conserved across vertebrate species, since it is evident in the model fish *Tetraodon*, in the model frog *Xenopus* and two healthy human tissues. This does not seem to hold in invertebrates, which actually exhibit distinct mechanisms of DNA methylation and extreme diversity [49, 50]. In general, across vertebrates, several characteristics of the sequences related to DNA methylation, like the CpG density, are conserved around the TSSs. These characteristics slightly differ for fish, where there is higher CpG density, but still signatures of CGI promoters [47]. Nevertheless, even though the CpG density differs, conserved mechanisms of regulation are present as reflected in this study. Overall, there seems to be a conserved role for the DNA methylation of the first intron across tissues, vertebrates and developmental stages.

The classic promoter methylation–gene expression model seems to hold only in extreme cases and specific genes, while a “triple-inverse” model was suggested, where the methylation of the promoter and the gene body exerts separate influences on gene expression [51]. The variability of DNA methylation in the first intron from 0 to 100% independently of the expression level could be linked to the more variable and larger length of the intron and suggestive of a more complex role of this methylation. In our case, a general pattern would be a negative association of the methylation of the first intron with gene expression in the majority of genes, which is clearest at the extremes of the gene expression range. Nevertheless, subclasses of genes escape this rule and exhibit positive correlations. Indeed, at the genome-wide level there are clusters of genes, each one showing different DNA methylation patterns associated with gene expression [38, 52]. DNA methylation in positive correlation with gene expression has been suggested to appear either as cause or consequence of transcription [53]. Our results also suggest a permissive state of gene expression linked with low methylation, but not a linear inhibitory link with high methylation. These observations are in support of the increasing evidence of a far more complex relationship of the epigenetic modifications—including histone and DNA modifications and noncoding RNAs—with gene expression at a spatiotemporal scale [52].

The function of the inverse relationship between DNA methylation of the first intron and gene expression could be partially explained by the presence of intronic enhancers interacting with the promoters of their corresponding genes. Indeed, the intron-mediated enhancement is a well-described phenomenon [48, 54, 55]. Silencing of intragenic enhancers is considered to play a role even more significant than promoter methylation in the silencing of their target genes [7]. In addition, intronic enhancers present tissue-specific methylation status associated with gene expression [56, 57]. Our results also revealed enriched TF-binding motifs common between tissues. Moreover, the unmethylated CpGs tend to be located closer to the beginning of the first intron and associated with higher gene expression levels, while the methylated CpGs tend to be further downstream and associated with lower gene expression levels. Therefore, the methylation status of the CpGs at shorter or greater distances from the beginning of the first intron which belong to a TF-binding motif is indicative of the gene expression level. Taken together, these results further support a regulatory role for the DNA methylation in the first intron region, although further experiments are needed to demonstrate the mechanistic relationships at the functional level.

tDMRs located in the whole gene showed both positive and negative correlation with gene expression as in human tissues [11]. Here, in addition to confirm this in a phylogenetically distant species, we partition the genomic localization of tDMRs in three important gene features: promoter, first exon and first intron. tDMRs are distributed across all these gene features and exhibit both directions of correlation, but no enrichment of positive or negative correlation depending on the location. This is in accordance with the latest findings in human tissues [11] and in contrast to the standard model of gene regulation by DNA methylation. However, in the first intron, there are more tDMRs, in agreement with our finding of the importance of the first intron in the regulation of gene expression by DNA methylation. In human macrophages after bacterial infection, the majority of gene body DMRs were also located in the first introns of genes [58]. Taken together, these results suggest an overlooked role for the DNA methylation of the first intron in the tissue-specific regulation of gene expression.

We used RRBS to assess DNA methylation and RNA-seq to measure the gene expression levels in the European sea bass genome which is one of the best annotated teleost genomes [31]. Nevertheless, the precision of the annotation of genes and their regulatory elements is not comparable to model species, like human or mouse. Therefore, we defined promoters as the region − 1000 kb upstream the predicted TSSs, as commonly arbitrarily defined [59–64], but without excluding the possibility of alternative TSSs or variable promoter lengths. The limitations of the sea bass genome annotation may influence also the gene expression data, where the analysis could only be performed at the gene level, based on the current annotation. RRBS allows for enrichment of the standard relevant parts of the genome for DNA methylation, e.g., promoters and CpG islands, and requires only a modest amount of sequencing [65, 66], making it a cost-effective alternative to WGBS which is considered generally inefficient since only 20–30% of the reads provide relevant information [67, 68]. Our RRBS results, including the genome representation and the actual methylation values, are comparable to other teleost fish, like the stickleback [69], the Atlantic salmon [70] and the zebrafish [71]. Regardless of the limitations of this study related to the main species in question and the techniques used, our key results were confirmed in other vertebrate species, corroborating the general trends shown in the sea bass, even accepting the possibility that some genes may be not well annotated.

## Conclusions

Our integrative analyses clearly reveal the important and conserved role of the methylation level of the first intron and its inverse association with gene expression regardless of tissue and species. Notably, the first intron exhibits a tissue-independent enrichment for TF-binding motifs and the methylation of the CpGs they contain is indicative of the gene expression level. Furthermore, the first intron presents a higher number of tDMRs than other gene features, suggestive of a regulatory role in tissue-specific expression. These findings not only contribute to our basic understanding of the epigenetic regulation of gene expression but also identify the first intron as an informative gene feature regarding the relationship between DNA methylation and gene expression where future studies could be focused, e.g., for the design of target sequences or for the analysis of genome-wide data throughout the region downstream the TSSs.

## Methods

### Animals

Wild European sea bass (*Dicentrarchus labrax*) adults with body weight = 1000 ± 109.5 g (mean ± SEM), standard length = 39.3 ± 1.4 cm and gonadosomatic index = 0.076 ± 0.009, the latter calculated as in [72], were captured by speargun at the Montgrí, Medes Islands and Baix Ter Natural Reserve (NE Spain) during the non-reproductive season (June 2013). Since the fish were caught in the wild, even if they were size-matched, they may have shown variation due to age, status or environment. Therefore, in further DNA methylation and gene expression analyses, biological variation was taken into account. Tissues were dissected immediately upon capture and stored in RNAlater^®^ (ThermoFisher Scientific).

### RNA isolation

Total mRNA was isolated from testis and muscle of five fish. Tissues were removed from RNAlater^®^, dried, immersed into TRIzol^®^ Reagent (ThermoFisher Scientific) and homogenized by the Polytron PT 1200 CL (Kinematica AG). RNA extraction was performed according to the manufacturer’s instructions. RNA was quantified by the Qubit^®^ RNA BR Assay Kit (ThermoFisher Scientific), and RNA quality was evaluated by the Agilent RNA 6000 Nano Kit (Agilent). Samples with RNA integrity number (RIN) > 8 were used for library construction.

### RNA-seq

The libraries were prepared using the mRNA-Seq sample preparation kit (Illumina Inc., Cat. # RS-122-2001x2) according to the manufacturer’s protocol. Briefly, 0.5 μg of total RNA were used for poly-A-based mRNA enrichment selection using oligo-dT magnetic beads followed by fragmentation by divalent cations at elevated temperature resulting into fragments of 80–250 nt, with the major peak at 130 nt. First-strand cDNA synthesis by random hexamers and reverse transcriptase was followed by the second-strand cDNA synthesis. Double-stranded cDNA was end-repaired and 3′-adenylated, and the 3′-“T” nucleotide at the Illumina adapter was used for the indexed adapters ligation. The ligation product was amplified using 15 PCR cycles. Each library was sequenced using the TruSeq SBS Kit v3-HS, in 76-bp paired-end mode on an Illumina HiSeq 2000 instrument following the manufacturer’s protocol. Images from the instrument were processed using the manufacturer’s software to generate FASTQ sequence files.

### RNA-seq analysis

RNA-seq reads were aligned with the GEMtools RNA-seq pipeline v1.7 (http://gemtools.github.io), which is based on the GEM mapper [73]. The pipeline aligns the reads in a sample in three phases, mapping against the reference genome (dicLab v1.0c, Jul. 2012), against a reference transcriptome (COMBINED ANNOTATION track) and against a de novo transcriptome, generated from the input data to detect new junction sites. The sea bass genome used here is one of the best in silico annotated fish genomes [31]. After mapping, all alignments were filtered to increase the number of uniquely mapped reads. The filtering criteria included a minimum intron length of 20 bp, a maximum exon overlap of 5 bp and a filter step against a reference annotation checking for consistent pairs and junctions where both sites align to the same annotated gene. The libraries’ statistics including the number of raw reads and the average reads aligned can be found in Additional file 2. The same pipeline was used to quantify expression at the gene level. Similarity across RNA-seq samples was investigated with principal component analysis (PCA; Additional file 1: Fig. S8A), where the first principal component separated the samples by tissue type and explained almost 96% of the variance. One muscle sample was excluded from further analysis since it was clearly an outlier in the quality clustering. The variation in gene expression values was higher and with more extreme values in muscle (Levene’s test; *p* < 0.001), whereas the testis had higher expression median (Mood’s median test; *χ*^2^ = 3372.7, *p* < 0.001; Additional file 1: Fig. S9A). Subsequently, the TMM method [74] was used for gene expression normalization, which takes into account not only library size (sequencing depth) of the samples but also the composition of the RNA population. The EdgeR robust method [75] was used for differential expression analysis. Genes with *p* adjusted < 0.05 were considered significant. Positive log_2_-transformed fold change (FC) indicates up-regulation in testis, and negative log_2_FC indicates down-regulation in testis. There were 9449 up-regulated genes and 6220 down-regulated genes in testis compared to muscle (FDR < 0.05). Furthermore, most of the genes expressed in both tissues had higher expression levels in the testis than in the muscle (Additional file 1: Fig. S9B). Tissue-specific genes were considered the genes that were expressed in only one of the two tissues, regardless of the actual expression level. Approximately 20 and 2000 genes accounted for half of the number of reads mapped in the muscle and testis, respectively (Additional file 1: Fig. S9C). However, the testis-specific genes had lower median expression (Mood’s median test; *χ*^2^ = 32.282, *p* < 0.001) than the muscle-specific genes (Additional file 1: Fig. S9D). These results confirmed that the testis and muscle constitute two tissues with very different transcriptomic complexity and validated our choice of tissues for the purposes of this study.

### DNA isolation

Genomic DNA was extracted by phenol/chloroform/isoamyl alcohol (PCI) from 3 of the samples of testis and muscle used to prepare RNA-seq libraries from a fragment contiguous to the one used for RNA extraction. DNA was extracted also from liver and spleen of one of the same fish. In brief, tissue samples were dried out from RNAlater^®^ and immersed into digestion buffer (0.1 M NaCl, 10 mM Tris–HCl, 1 mM EDTA pH 8, 0.5% SDS), and proteins were digested by 1 μg of proteinase K (Sigma-Aldrich) and RNA by 0.5 μg of ribonuclease A (PureLink RNase A; Life Technologies). DNA was precipitated by 95% ethanol, eluted in Milli-Q^®^ water (Merck, Millipore) and cleaned up with 2× AMPure XP beads (Beckman Coulter) to ensure purity. DNA was quantified three times by independent means, being by ND-spectrophotometer (NanoDrop Technologies) or Qubit™ fluorometric quantitation (ThermoFisher Scientific), each time followed by dilutions with nuclease-free water in order to normalize DNA quantities across samples.

### RRBS libraries preparation

RRBS libraries were prepared as in Klughammer et al. [76]. One hundred nanograms of genomic DNA was digested by 20 units of *Msp*I (NEB) overnight at 37 °C. Five units of Klenow fragment (3′ → 5′exo-; NEB) and dNTP mix (final concentration: 300 μM dATP, 30 μM dCTP and 30 μM dGTP) were added to the reaction. End fill-in was performed for 20 min at 30 °C, A-tailing for 20 min at 37 °C and inactivation of the enzyme for 20 min at 75 °C. Ligation of Illumina TruSeq Adapters v2 was performed by Quick Ligase (NEB) for 20 min at 25 °C, followed by heat inactivation of the enzyme for 10 min at 65 °C. Libraries were size-selected by 0.75× 1:5 diluted AMPure XP beads, quantified by qPCR, pooled based on qPCR values and cleaned up with 2.5× 1:5 diluted AMPure XP beads. Samples were subjected to bisulfite conversion using the EZ DNA Methylation-Direct kit (Zymo Research) with 0.9x CT Conversion Reagent, 20 cycles of 95 °C for 1 min and 60 °C for 10 min and desulphonation time extended to 30 min. Libraries were enriched by the PfuTurbo Cx HotStart Polymerase (Agilent Technologies) with the following cycling parameters: 95 °C for 2 min, followed by the optimal number of cycles of 95 °C for 30 s, 65 °C for 30 s and 72 °C for 45 s, and a final step at 72 °C for 7 min. The optimal number of cycles for the enrichment PCR was calculated based on qPCR values. A final clean-up step was performed by 1x AMPure XP beads. The quantity of the libraries was measured by Qubit High Sensitivity assays (ThermoFisher Scientific), and the quality was evaluated by Experion DNA 1 k assays (BioRad). Sequencing of RRBS libraries was performed on an Illumina HiSeq 2000 platform in 50-bp single-end mode.

### DNA methylation analysis

RRBS raw reads were quality trimmed by the Trimmomatic v. 0.32 [77] using a sliding window trimming with window size 4 and required quality 15, an adaptive quality trimming with the target length set at 20 and the strictness at 0.50 and a minimum read length of 18 bp. Trimmed reads were aligned to the reference genome of sea bass using BSMAP v. 2.90 [78] in RRBS mode requiring a minimum coverage of 5 reads. Methylation calling was performed by the *methratio.py* python script that accompanies BSMAP. The bisulfite conversion rate was calculated using the *bsrate* script of the MethPipe pipeline v. 3.4.3 [79]. In brief, the RRBS libraries showed a mean of 40,342,296 reads, a mean alignment rate of 81.26%, 1,122,487 covered CpGs, a mean fold coverage of 60.16 and 99.1% of bisulfite conversion ratio. The statistics of the libraries per sample including the number of raw reads, the alignment rates, the number of covered cytosines and the bisulfite conversion ratio can be found in Additional file 2. All subsequent bioinformatics analyses were performed using R v. 3.4.1 and Rstudio v. 1.0.143 [80, 81] and Bioconductor packages [82], unless stated otherwise. The package *methylKit* v. 1.2.0 [83] was used for DNA methylation analysis. Called bases with less than 10 reads or more than the 99.9th percentile of coverage distribution were filtered out. Coverage values were normalized as by default and bases were united in order to retain the ones that were covered in all samples which were 529,070. Pairwise comparisons of RRBS DNA methylation values for testis and muscle showed good correlation between biological replicates within each tissue and higher for testis (Pearson’s correlation scores: testis ≥ 0.97; muscle ≥ 0.83, Additional file 1: Fig. S8B). Overall, DNA methylation levels were similar between the two tissues and showed a strong positive correlation (Pearson’s product-moment correlation = 0.95, *p* value < 0.001; white to blue scale in Additional file 1: Fig. S10A). Differentially methylated cytosines (DMCs) between tissues were defined as CpGs with more than 15% methylation differences and *q* value < 0.01 after applying logistic regression using the SLIM method for *p* value adjustment. Positive values indicate hyper-methylation in testis, and negative values indicate hypo-methylation in testis. Among the 500 top-differentially methylated CpG (DMC) sites, there were ~ 2.8 times more hyper-methylated CpGs in the testis than in the muscle (Additional file 1: Fig. S10B), while there were no CpG sites with > 90% methylation in muscle and < 10% methylation in the testis (green to red scale in Additional file 1: Fig. S10A). Differentially methylated regions (DMRs) between tissues were identified using the weighted optimization algorithm for empirically based DMRs of the package *edmr* v. 0.6.4.1 [84] with default parameters, except for DMC differences cutoffs which were set to 15% and DMR differences cutoffs set to 15%. We used a 15% cutoff for defining differential methylation after exploratory analyses with variable thresholds, since it represented a good compromise between robust differential methylation and retention of potentially interesting loci.

### Combined analysis of DNA methylation and gene expression

A BSgenome package [85] was created for use when required using the full sea bass genome and masks from the UCSC server (dicLab v1.0c, Jul. 2012). Annotations of gene features were based on the COMBINED ANNOTATION track. Promoters were defined as 1000 bp upstream the in silico annotated transcription start sites (TSSs) from the COMBINED ANNOTATION track. The DNA methylation levels of gene features were calculated by averaging the methylation values per gene feature. Genomic overlaps of features were identified using the GenomicRanges (v. 1.28.4) package [86].

The vioplot (v.0.2) package was used for visualizing methylation data by expression decile [87]. For positive and negative correlations of methylation differences and gene expression, first we identified the DMRs located in genomic regions encompassing the whole gene bodies and 4 kb upstream from the TSSs or downstream of the 3' UTR. Then, DMRs overlapping with promoters, first exons or first introns were identified. Only genes with log_2_ FC > |1.5| and FDR < 0.05 were considered as differentially expressed. Enrichment of transcription factor (TF) binding motifs was performed using the ame tool v. 4.9.1 [88] of the MEME suite [89] using as input the JASPAR CORE 2016 vertebrates database [90] and shuffled input sequences as controls for enrichment. AME shuffles the input sequences while preserves the dinucleotide frequencies. TF-binding motifs were considered enriched if *p* value < 0.001 after Bonferroni correction of multiple Fisher’s exact test. The sequencing scoring method was the average odds score and the input sequences contained ± 50 bp from the CpGs of the first introns. This distance was chosen to encompass the maximum TF-binding motifs length (31 nucleotides [36]) and an arbitrary + 20 nucleotides more. Four enriched TF-binding motifs common between muscle and testis that contained CpG sites were selected for screening in the sequences of the first introns using the fimo tool v. 4.9.1. CpGs present in these sequences were classified as unmethylated if their methylation was below the first quartile of the distribution and as methylated if their methylation was above the third quartile of the distribution. The relative distance of these CpGs from the start of the first intron was calculated as the distance from nucleotide 0 (bp)/width of the intron (bp). Detection of TF-binding motifs inside the sequences of tDMRs in gene bodies  4 kb upstream from the TSS or downstream of the 3’ UTR was performed using the fimo tool v. 4.9.1 to scan for the motifs of the JASPAR CORE 2016 vertebrates database. Only TF-binding motifs with a *q* value < 0.01 for both muscle and testis were considered.

### Other species data

WGBS-seq and RNA-seq data of whole *Tetraodon nigroviridis* and of muscle tissue of *Ciona intestinalis* were obtained from the study [20] (NCBI Gene Expression Omnibus [33, 91] with accession number GSE19824). WGBS-seq from gastrula stage 10.5 and RNA-seq data from gastrula stage 11 of *Xenopus tropicalis* were obtained from the study [34] (GEO with accession number GSE67974). WGBS-seq and RNA-seq data of normal human lung and liver were obtained from the study [35] (GEO with accession number GSE70091). For the human data, 3 replicates were available; therefore, after excluding positions with less than 10 reads coverage from the WGBS data, the methylation per position was calculated as 100*methylated_read_count/total_read_count and averaged for the three replicates. Gene annotations were read with the readTranscriptFeatures function of the genomation v.1.8.0 package [92], and the first intron was selected. The methylation per gene feature was calculated as the average of CpGs covered in each gene feature. Expressed genes (cpm > 0) with methylation in the first intron were ordered, split in deciles according to their expression levels and plotted using vioplot.

### Statistical analysis of the data

Statistical analyses of the data were performed by R v. 3.4.1 and Rstudio v. 1.0.143 [80, 81]. The association of DNA methylation for pairs of gene features was calculated as the odds ratios: (N_00_ × N_11_)/(N_01_ × N_10_), where N_00_ = gene feature (GF) 1 < 10% and GF2 < 10%, N_11_ = GF1 > 90% and GF2 > 90%, N_01_ = GF1 < 10% and GF2 > 90% and N_10_ = GF1 > 90% and GF2 < 10%. The quantification of the strength of the association by odds ratio was chosen as done before for the same purpose [9]. The Wald approximation was used to calculate the confidence intervals at alpha = 0.001.

Correlations between DNA methylation data were measured using Pearson’s product-moment correlation coefficient. Correlations between DNA methylation and gene expression were measured using Spearman’s rank correlation coefficient because the relationship of DNA methylation with gene expression data is not necessarily expected to be linear. To compare the medians of gene expression between the two tissues, the Mood’s median test was used. Homogeneity of variances was checked by Levene’s test. To compare that variance of DNA methylation in gene features between tissues and expression deciles, an ANOVA on the residuals followed by Tukey’s honest significant differences was performed. For pairwise comparisons of DNA methylation values between the extreme expression groups, the Wilcoxon rank sum test with continuity correction was used, after removing outliers as defined by Tukey fences (values below *Q*_1_ − 1.5(*Q*_3_ − *Q*_1_) or above *Q*_3_ − 1.5(*Q*_3_ − *Q*_1_)). The effects of relative distance and methylation status on gene expression using analysis of covariance after checking for normality of the residuals.

## Additional files

**Additional file 1.** Supplementary Figures and Tables.

**Additional file 2.** RRBS and RNA-seq libraries basic statistics. This file includes the raw number of reads, the alignment rates and other basic statistics of the NGS libraries prepared for this study.
