# Supplementary material for: Consistent inverse correlation between DNA methylation of the first intron and gene expression across tissues and species
Source: Epigenetics Chromatin. 2018 Jun 29;11:37. doi: 10.1186/s13072-018-0205-1 (PMC6025724; doi:10.1186/s13072-018-0205-1)
Supplement: Supplementary file 1 — Additional file 1. Supplementary Figures and Tables. [file 13072_2018_205_MOESM1_ESM.docx]

**Consistent inverse correlation between DNA methylation of the first intron and gene expression across tissues and species**

Dafni Anastasiadi^1^, Anna Esteve-Codina^2,3^, and Francesc Piferrer^1*^

^1^Institute of Marine Sciences (ICM-CSIC), Passeig Marítim de la Barceloneta 37-49, 08003 Barcelona.

^2^CNAG-CRG, Centre for Genomic Regulation (CRG), Barcelona Institute of Science and Technology (BIST), Baldiri i Reixac 4, 08028 Barcelona.

^3^Universitat Pompeu Fabra (UPF), Barcelona.

**Supplementary Figures**


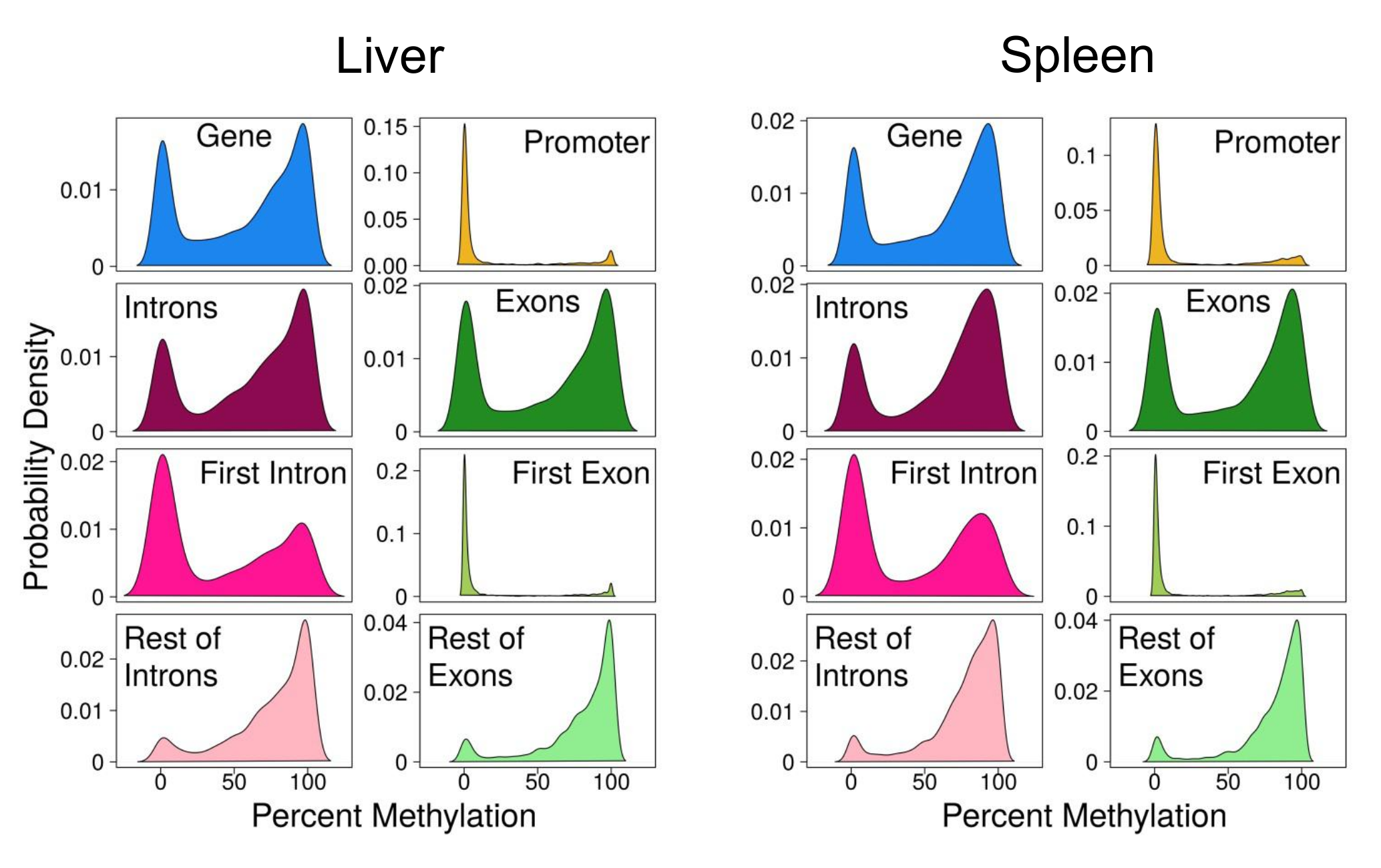


**Figure S1**. Distribution of DNA methylation in gene features in liver and in spleen. Kernel density plots for DNA methylation in genes, promoters (-1000 bp from the Transcription Start Site), all introns and all exons. Separation of exons in first exon and rest of exons and of introns in first intron and rest of introns.


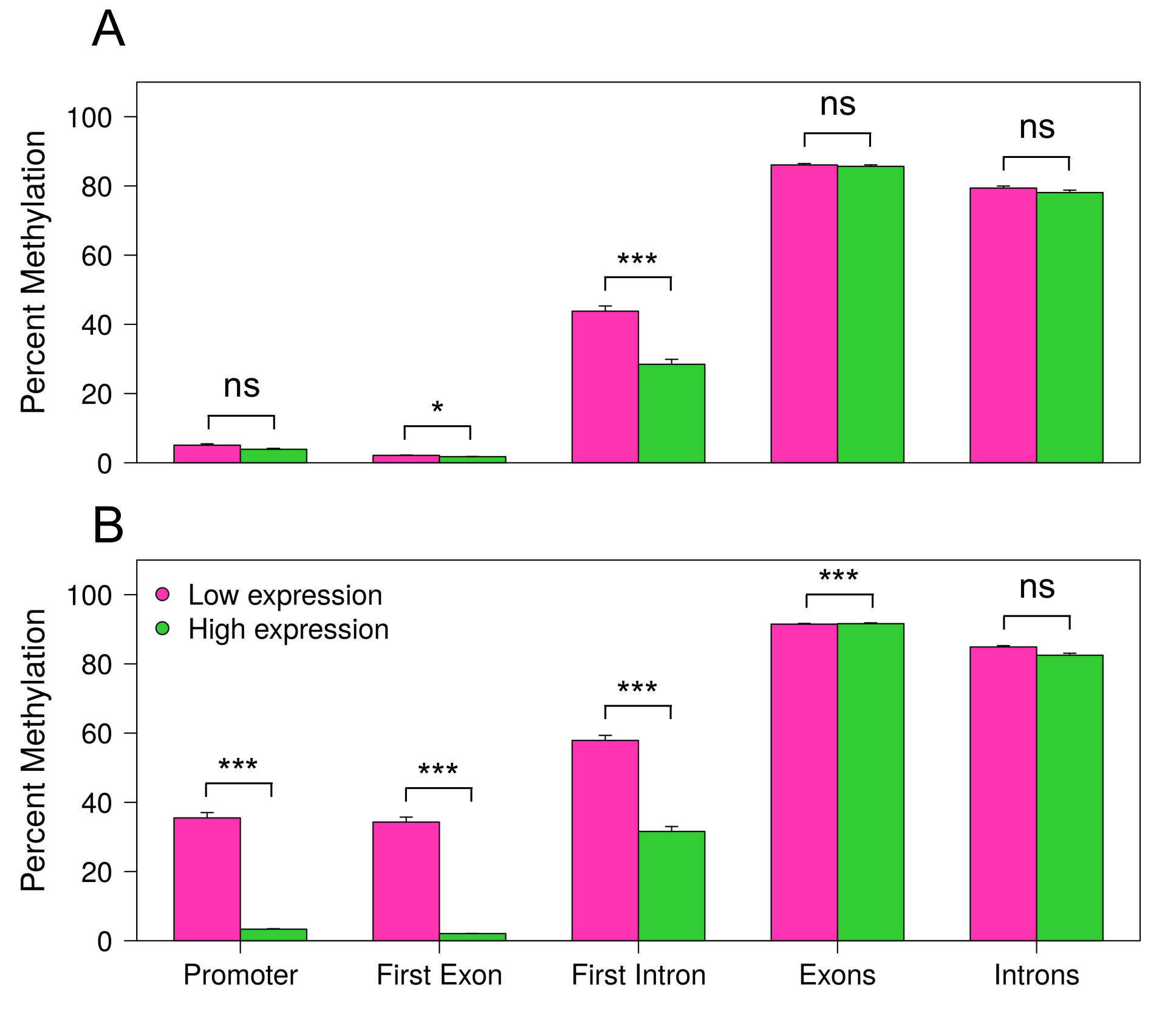


**Figure S2.** DNA methylation is shown per gene feature in muscle (A) and testis (B) for the genes that belong to the two first expression deciles (Low expression, pink) and to the last two expression deciles (High expression, green). Outliers were removed based on Tukey Fences (values below Q_1_-1.5(IQR) or above Q_3_-1.5(IQR)) and mean DNA methylation is shown with bars indicating the standard error of the mean. The Wilcoxon rank sum test with continuity correction was used to test for statistical differences of DNA methylation between lowly and highly expressed genes, which are reported with the following equivalence: ****p*<0.001, **p*<0.05, ns=not significant.


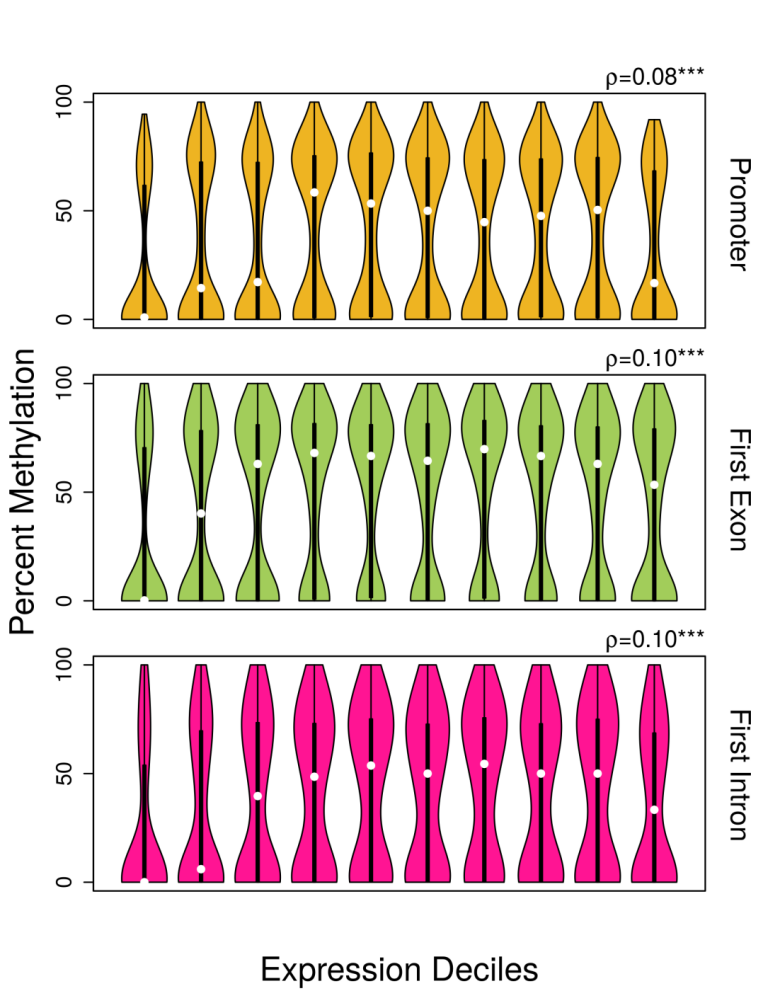


**Figure S3**. DNA methylation in gene features by expression deciles in *Ciona intestinalis*. Violin plots of DNA methylation in promoter (n=4090), first exon (n=3824) and first intron (n=6291) divided in deciles based on increasing ranking of gene expression measured as log_2_-transformed count per million (cpm) values. Box plots with rotated kernel density plots at both sides indicate the interquartile range and white central dots the median of the distribution. Correlations between DNA methylation and gene expression were measured using Spearman’s rank correlation coefficient (ρ) and the significance levels are reported as follows: ***=*p*<0.001.


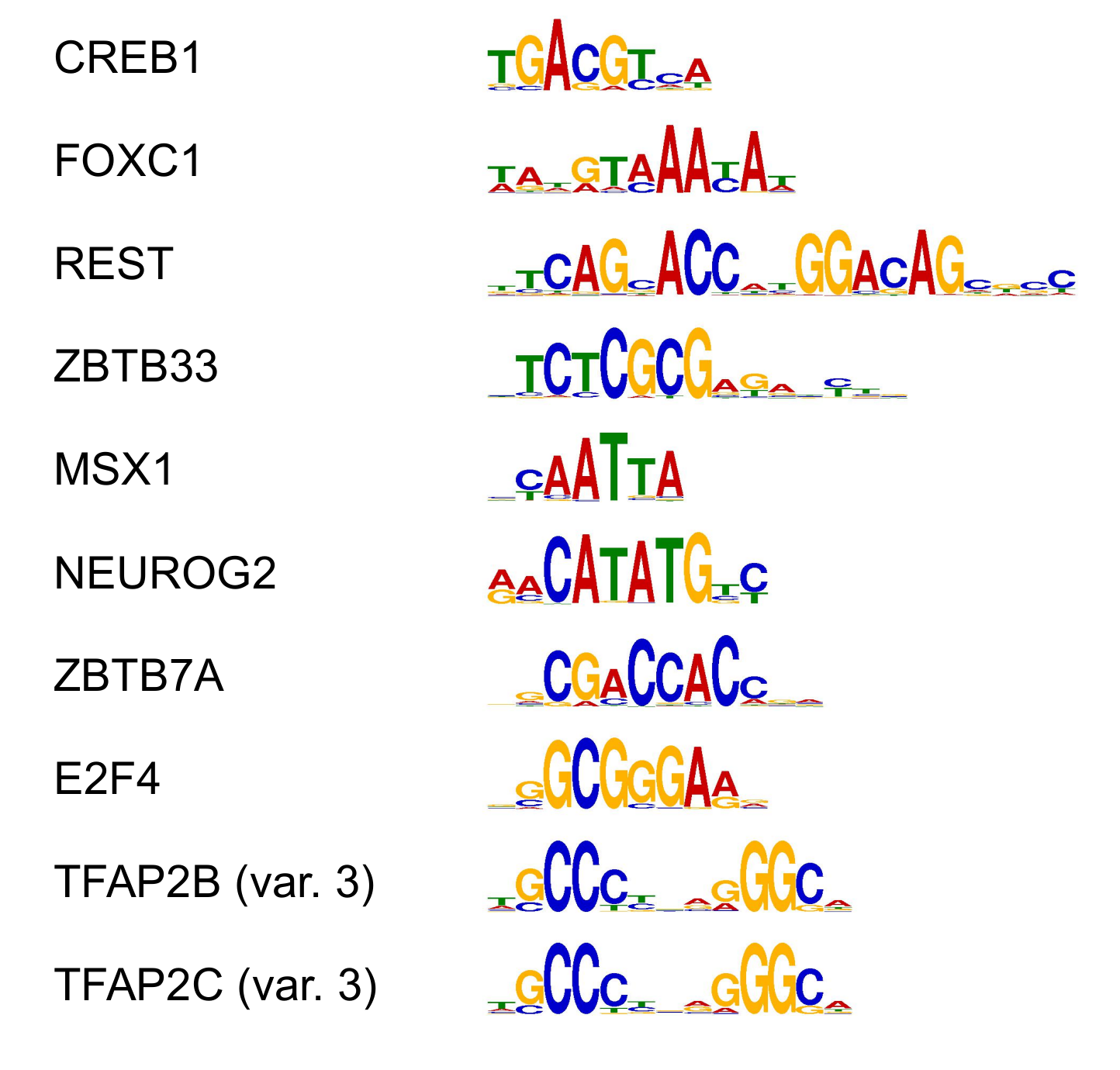


**Figure S4**. Binding sites of transcription factors (TF) enriched in the first introns. Logos of the consensus sequence based on the frequency matrix of nucleotides for TFs. These sequences are located in a distance of ±50 bp from the CpGs with DNA methylation values in the first intron of expressed genes and are common in muscle and testis. Motifs were considered enriched when the adjusted *p*-value after Bonferroni correction for multiple one-tailed Fisher’s exact tests was <0.001. Enrichment of TF-binding sites in the first introns was relative to control sequences as shuffled input sequences.


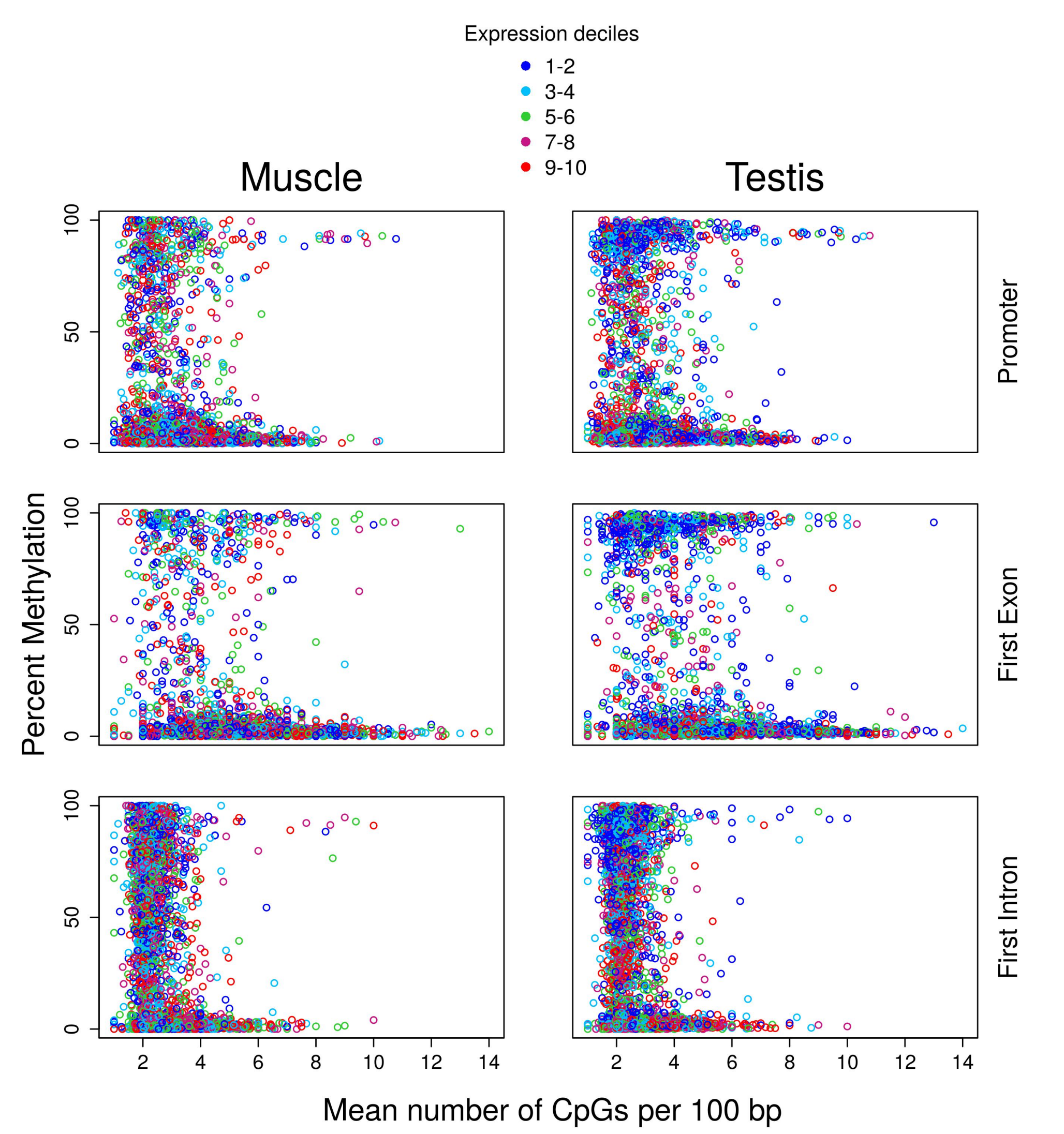


**Figure S5**. DNA methylation and CpG density per gene feature in muscle and testis. Each colored dot represents one gene with DNA methylation in the promoter (muscle, n=2745; testis, n=3345), first exon (muscle, n=3537; testis, n=4064), first intron (muscle, n=2801; testis, n=3122). Colors of dots follow the expression deciles based on increasing ranking of gene expression measured as log_2_-transformed count per million (cpm) values. CpG density was calculated after averaging the number of CpGs in interval windows of 100 bp in the sequences of interest for each gene.


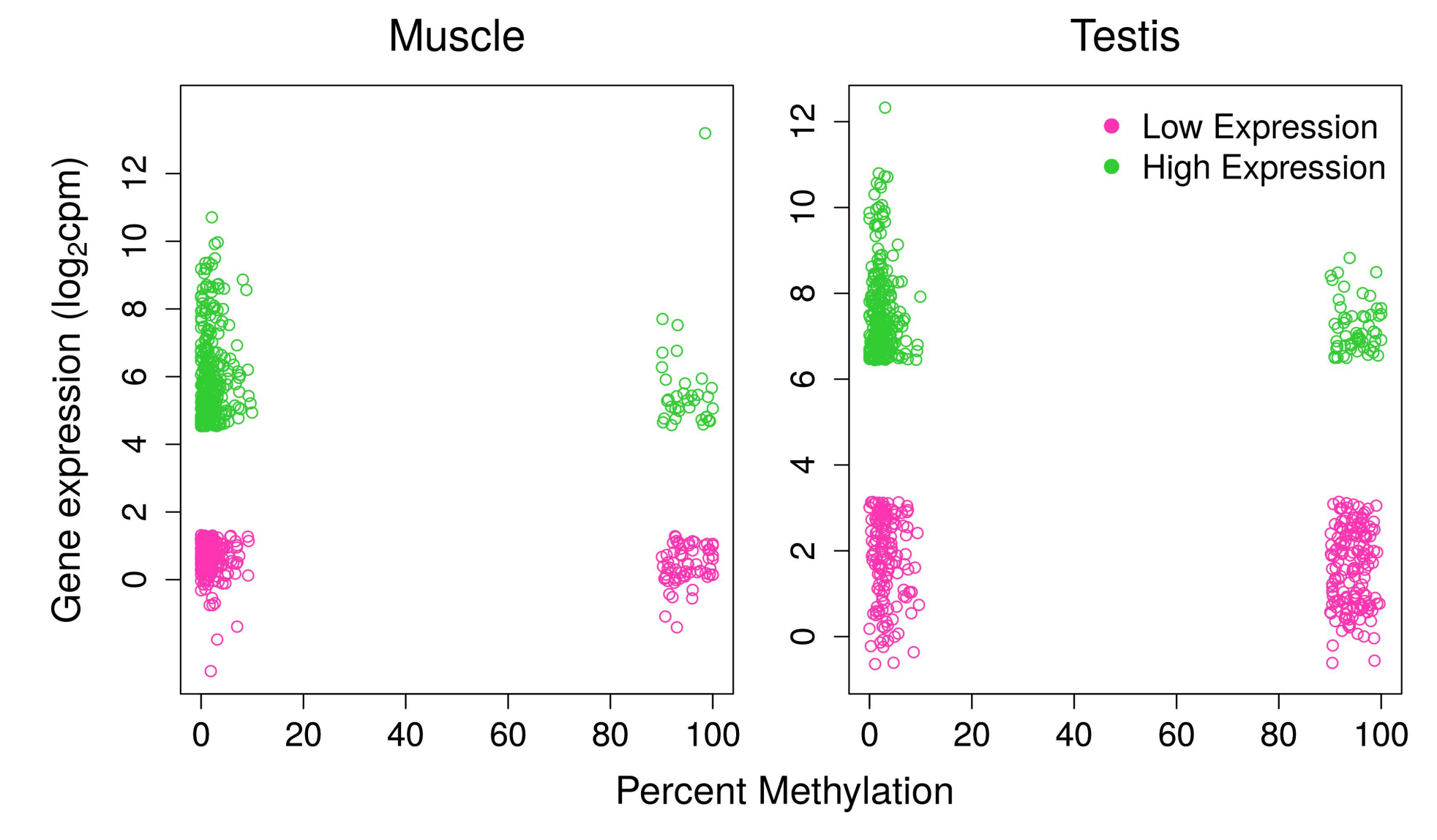


**Figure S6**. Gene expression and DNA methylation in the first intron of genes at the extremes of the expression range. Selected genes expressed in muscle and testis belong to the two first expression deciles (Low expression, pink) and to the last two expression deciles (High expression, green) and with DNA methylation in the first intron below 10% or above 90%.


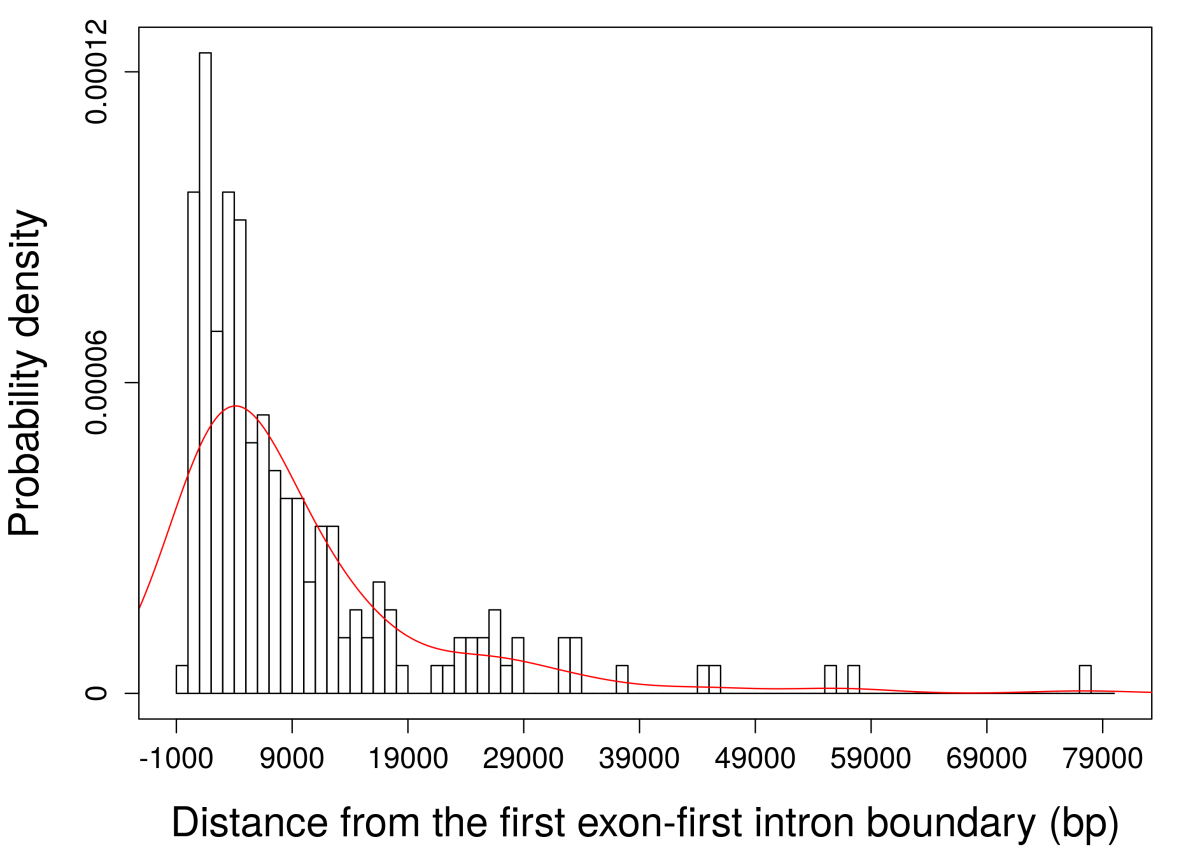


**Figure S7**. Distribution of tissue-specific differentially methylated regions (tDMRs) along the sequence of the first intron. The probability density is shown in the y-axis and the red line indicates shows the density curve. The middle point between the start and the end of each tDMRs was considered to estimate the distance from the first exon-first intron boundary.

**
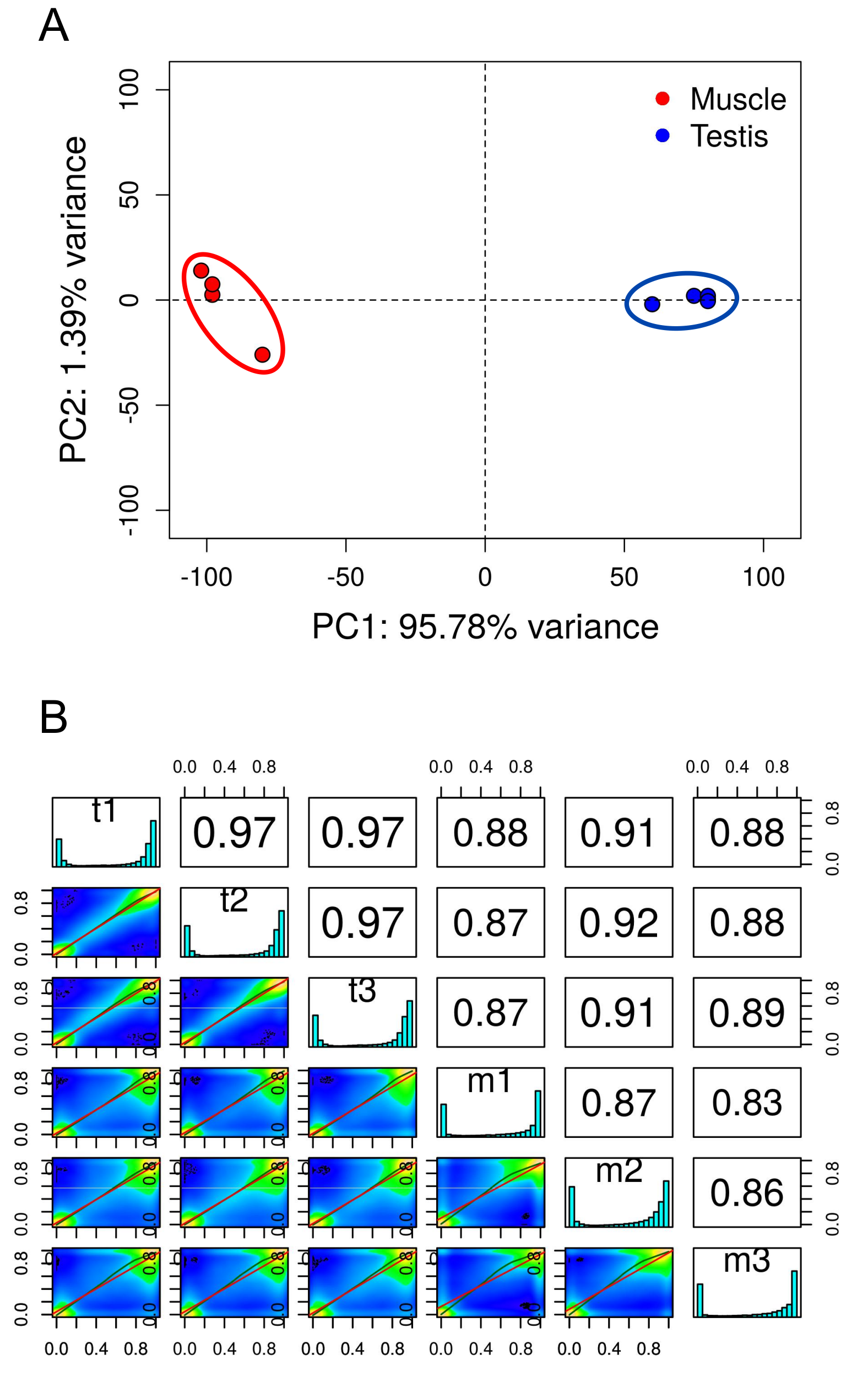
**

**Figure S8**. Similarity between biological replicates. A) Similarity between RNA-seq samples of muscle and testis as shown by Principal Component Analysis (PCA). B) Similarity between RRBS samples is shown for each pairwise comparison of DNA methylation values for testis and muscle. The distribution of DNA methylation values is indicated by scatterplots of percent methylation values for each pair and diagonal histograms per sample. In addition, Pearson’s correlation scores are given for each pairwise comparison.


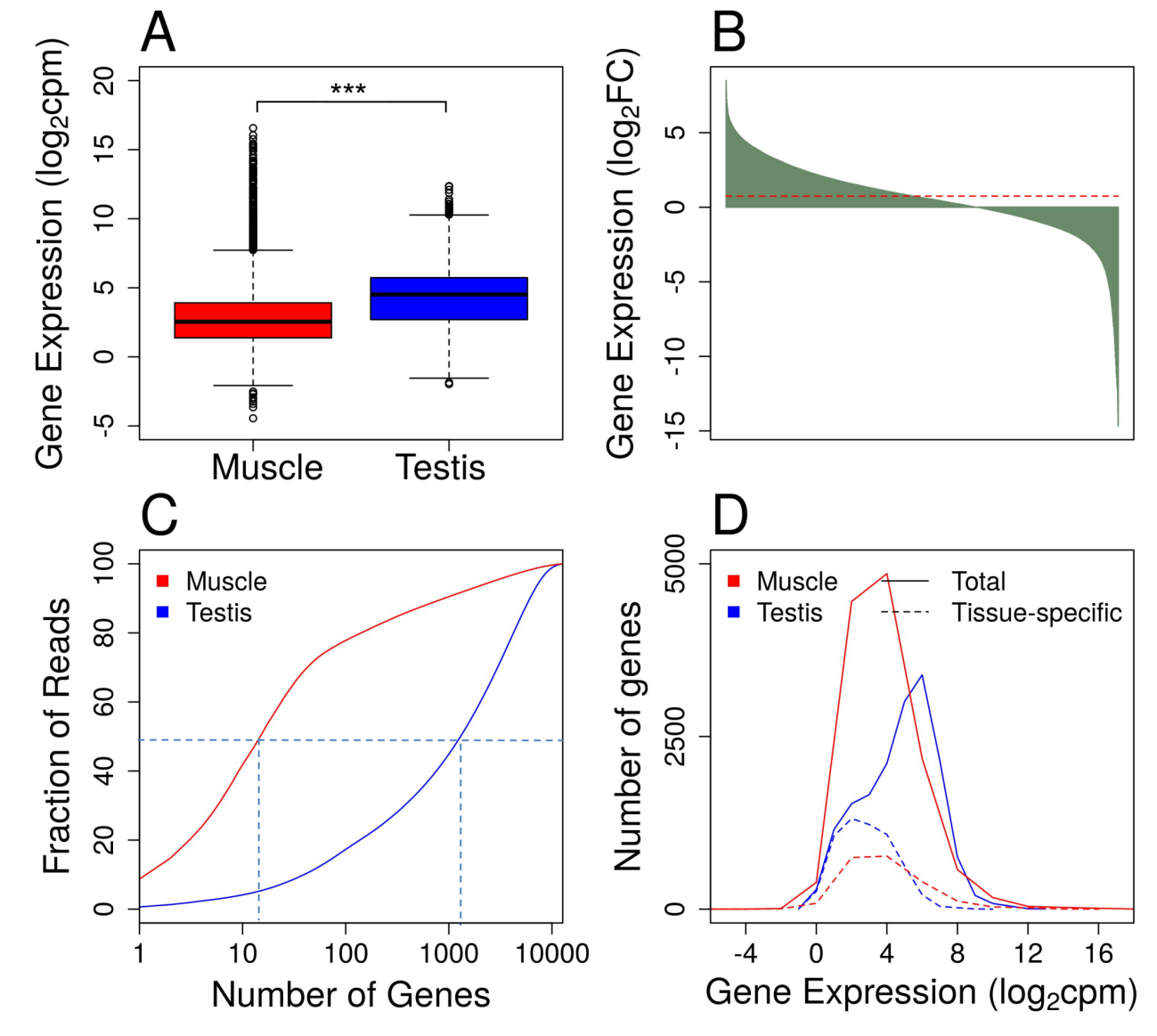


**Figure S9**. Gene expression overview. A) Boxplots of gene expression measured as log2-transformed count per million (cpm) values in muscle (red; n=12715) and in testis (blue; n=16380). The Mood’s median test indicates significant differences between the two tissues (*χ^2^*=3372.7, ***=*p*<0.001). The boxes show the interquartile distribution (IQR) of values, the upper whisker equals to the minimum value of max(x) or Q3 + 1.5 * IQR and the lower whisker equals to maximum value of min(x) or Q1 – 1.5 * IQR, the black lines indicate the median and the black dots expanding outside the boxes are considered outliers. B) Histogram of the ratio (testis/muscle) of log_2_-transformed fold change (FC) in decreasing order where positive values correspond to up-regulation in the testis and negative values correspond to down-regulation in the testis (n=10538). The dashed red line indicates the median of the distribution (median log_2_FC=0.745). C) Cumulative distribution of genes contribution to total number of reads in muscle (red; n=12715) and testis (blue; n=16380). The dashed lines indicate 50% contribution to total read counts and the associated number of genes. D) Distribution of total gene expression measured as log_2_-transformed count per million (cpm) values (continuous lines) and tissue-specific genes (dotted lines) in muscle (red; total, n=16380; tissue-specific, n=2177) and in testis (blue; total, n=16380; tissue-specific, n=5842).


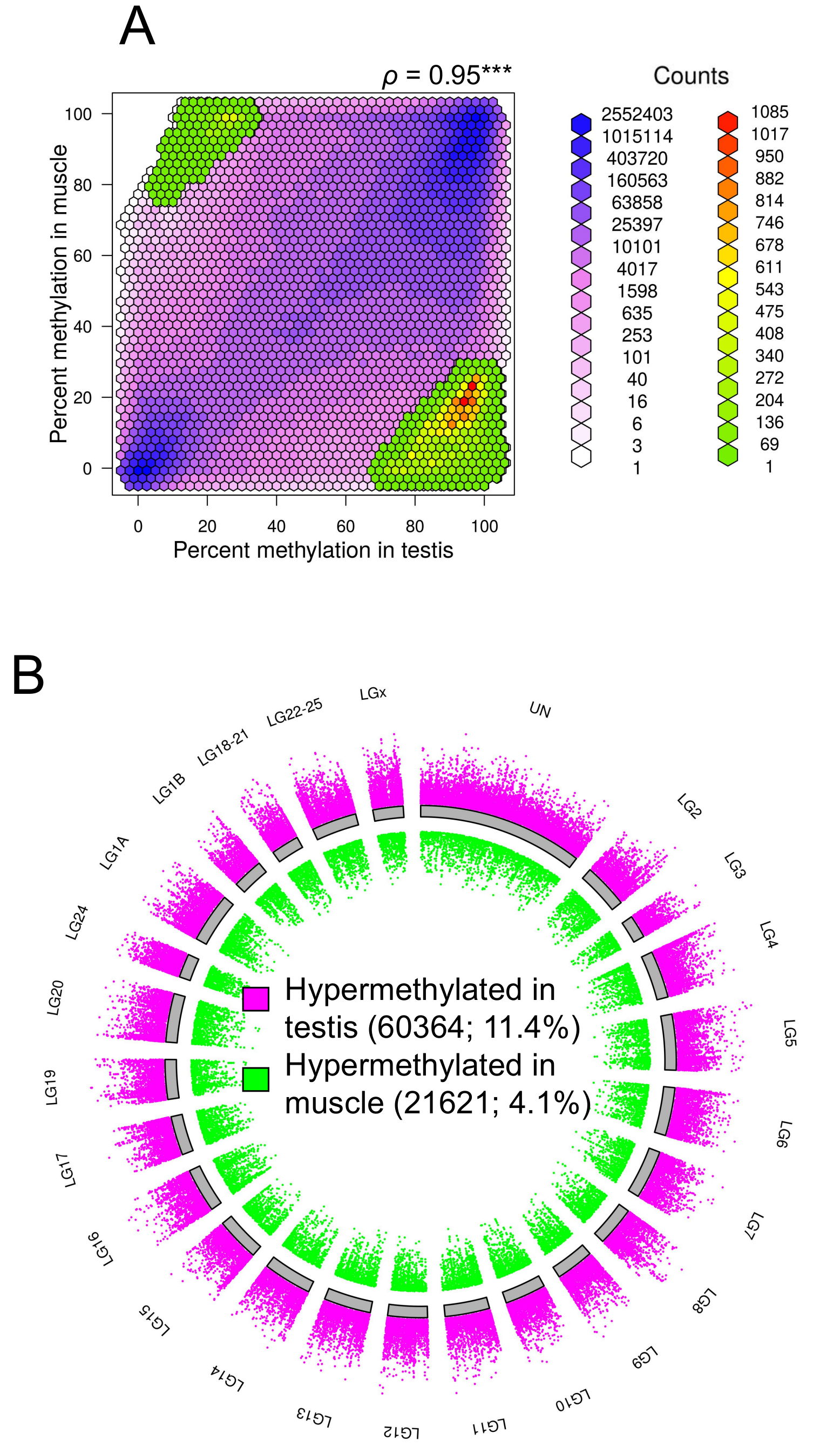


**Figure S10**. Overview of genome-wide DNA methylation in the European sea bass. A) DNA methylation levels in testis and in muscle. Scatterplots of DNA methylation in muscle vs. testis divided in 40 bins containing from 1 (white) to 2552403 (blue) data points (Pearson’s correlation *ρ* = 0.95, *p*<2.2^-16^; n=529070) and overlaid scatterplots of top 500 differentially CpGs divided in 40 bins containing from 1 (green) to 1085 (red) data points. B) Differentially methylated CpGs between testis and muscle across the European sea bass genome. Distribution of hypermethylated sites in testis (magenta; outer circle) and in muscle (green; inner circle) per chromosome (linkage group; LG).

**Supplementary Tables**

**Supplementary Table 1.** Correlation between DNA methylation of the first intron and gene expression depending on the length of the first intron

|  |  | Quartile 1 | Quartile 2 | Quartile 3 | Quartile 4 |
| --- | --- | --- | --- | --- | --- |
| Muscle | Min/max intron length (bp) | 45/1183 | 1186/3216 | 3232/10757 | 10766/289209 |
|  | Median intron length (bp) | 681 | 1866 | 5947 | 18746 |
|  | Median methylation | 1.87 | 2.92 | 43.21 | 63.14 |
|  | Spearman's rank correlation ρ | -0.12 | -0.09 | -0.1 | -0.06 |
|  | *p*-value | 0.001 | 0.021 | 0.006 | 0.096 |
| Testis | Min/max intron length (bp) | 45/1236 | 1239/3754 | 3771/11446 | 11469/163943 |
|  | Median intron length (bp) | 671 | 2132 | 6505 | 25989 |
|  | Median methylation | 2.76 | 4.44 | 59.93 | 73.77 |
|  | Spearman's rank correlation ρ | -0.29 | -0.31 | -0.16 | -0.17 |
|  | *p*-value | <2.2^-16^ | <2.2^-16^ | 8.61^-06^ | 2.05^-06^ |
